# Supplementary material for: MANNERS: A strategy for representation learning in multivariate datasets with high proportions of missing data
Source: Patterns (N Y). 2026 Apr 23;7(7):101543. doi: 10.1016/j.patter.2026.101543 (PMC13366519; doi:10.1016/j.patter.2026.101543)
Supplement: Document S1. Figures S1–S3 and Tables S1–S8 [file mmc1.pdf]

Patterns, Volume *7*

Supplemental information

MANNERS: A strategy for representation learning in multivariate datasets with high proportions of missing data

Louis Bellmann, Maximilian Nielsen, and Philipp Breitfeld

1 Supplemental Figures

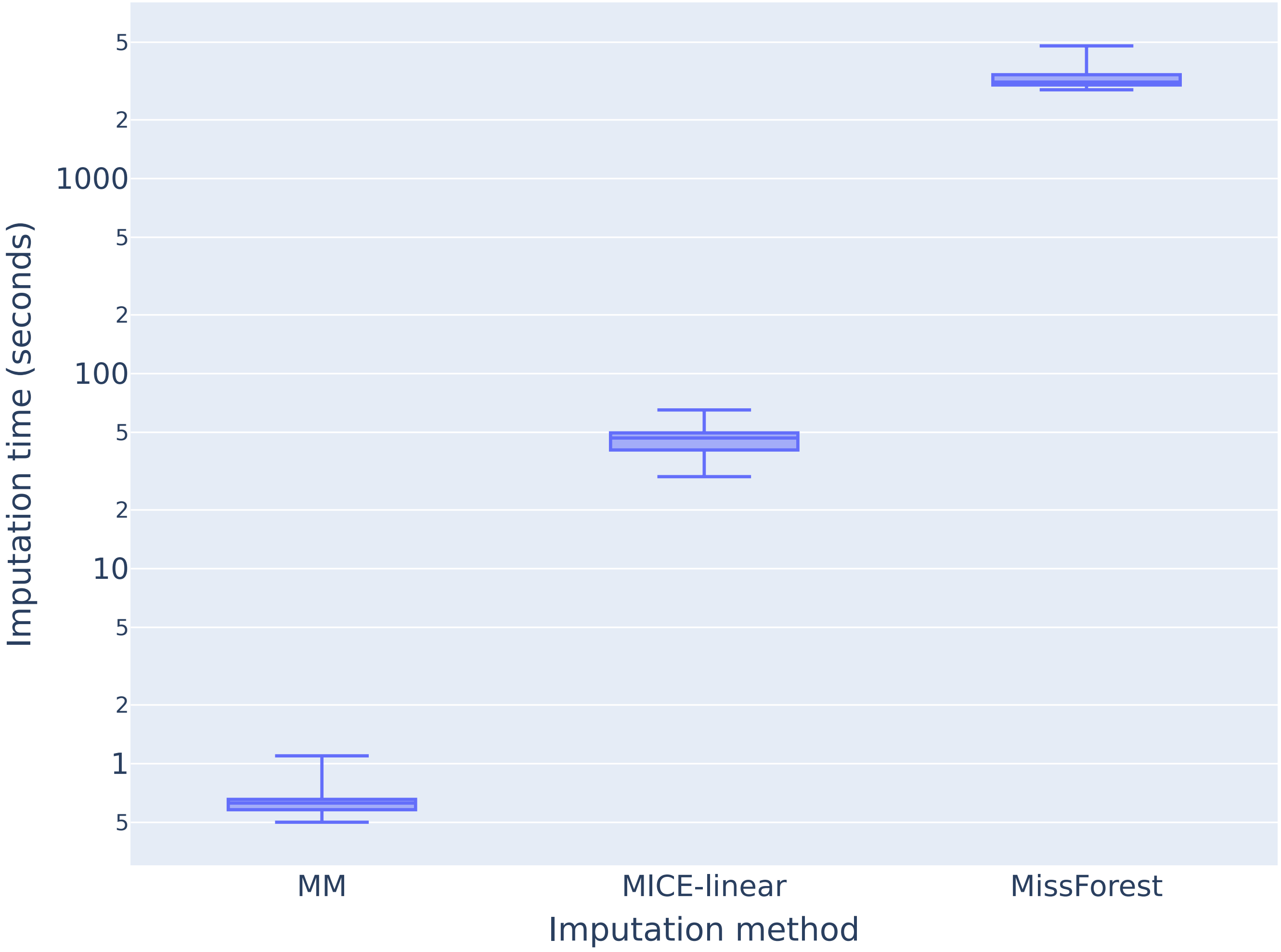

Figure S1: Runtimes per imputation method

Single-threaded runtimes per imputation method in seconds across 720 training runs in logarithmic units. Imputation methods are fitted to the training set and applied to training, validation and test set. The box represents the interquartile range (IQR), the line inside the box indicates the median, and the whiskers extend to the most extreme data points.

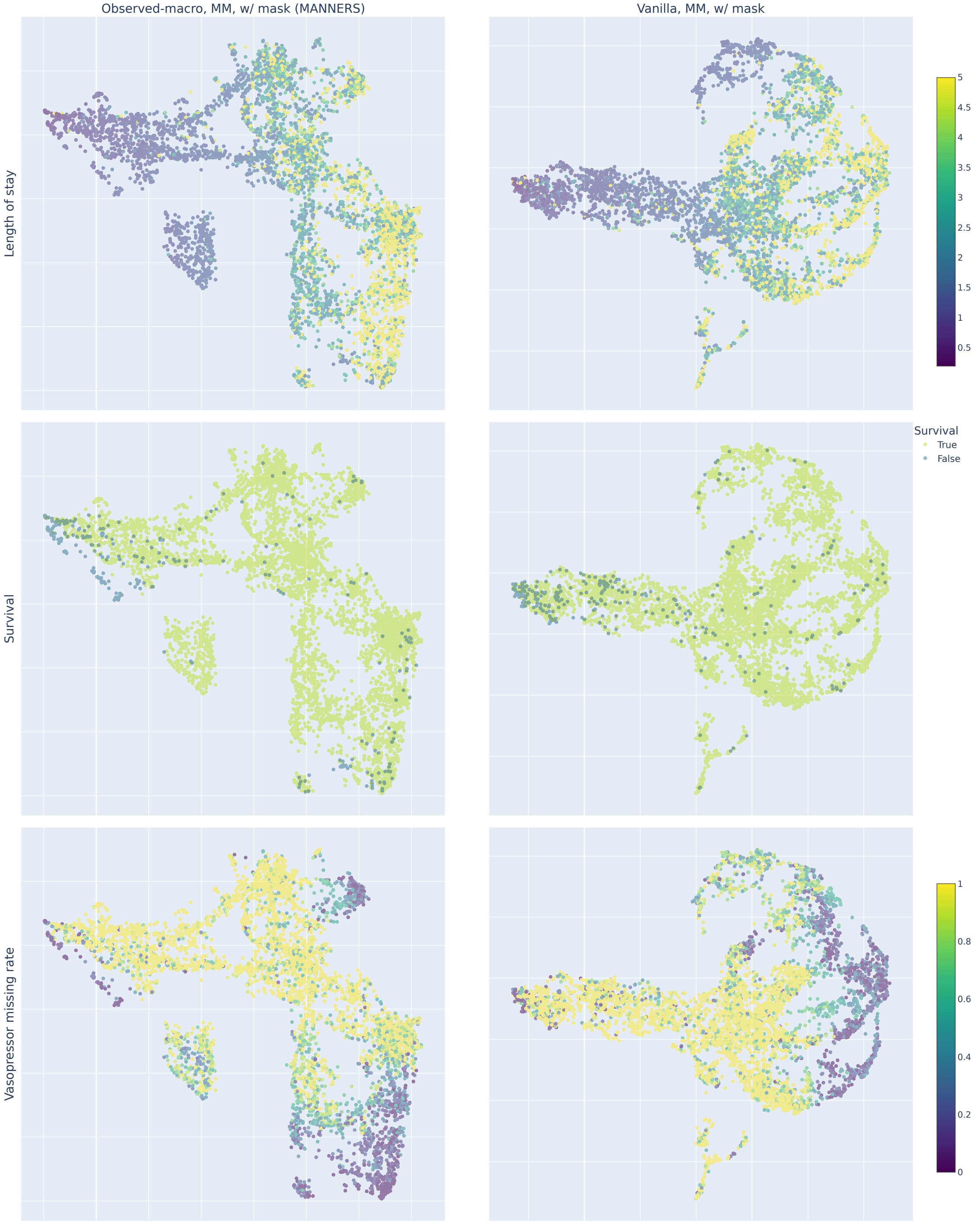

Figure S2: UMAP projections of latent representations

Test set representation projections into $\mathbb{R}^2$ on the first Monte Carlo cross-validation split for the MANNERS configuration with observed-macro loss normalization, MM imputation and mask encoding (left), and vanilla loss normalization, MM imputation and mask encoding (right). Projections are colored by length of ICU stay in fraction of days (top), survival during the first 48 hours (middle), and vasopressor missing rate (bottom).

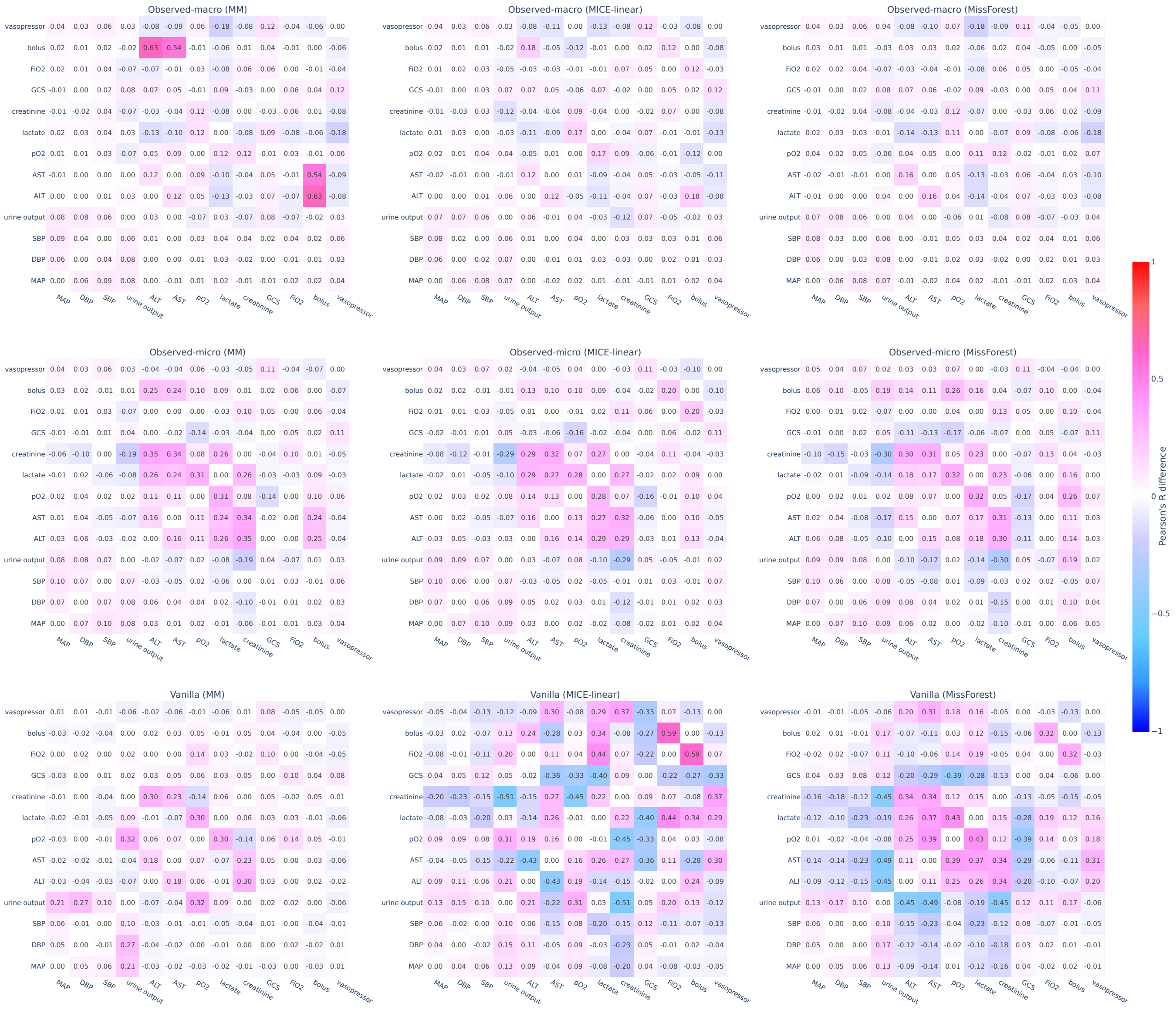

Figure S3: Correlation difference between synthetic and original dataset

Signed difference between Pearson correlation coefficients of the full original dataset and stacked synthetic datasets generated by 20 VAEs trained with observed macro (top), observed micro (middle), and vanilla (bottom) loss normalization strategies. Missing values were imputed with MM (left), linear MICE (middle), and MissForest (right) imputation techniques. All VAEs used missing mask encoding.

2 Supplemental Tables

Table S1: Missing mask reconstruction results

Loss normalization	Variable	MM	MICE-linear	MissForest
observed-macro	MAP	.996 (.0)	.995 (.0)	.995 (.0)
	DBP	.997 (.0)	.996 (.0)	.996 (.001)
	SBP	.997 (.0)	.996 (.0)	.996 (.001)
	urine output	.961 (.003)	.96 (.003)	.962 (.004)
	ALT	.995 (.001)	.995 (.001)	.994 (.001)
	AST	.995 (.001)	.996 (.001)	.995 (.001)
	pO2	.977 (.003)	.976 (.002)	.973 (.003)
	lactate	.977 (.003)	.977 (.002)	.975 (.003)
	creatinine	.991 (.002)	.99 (.003)	.99 (.002)
	GCS	.966 (.003)	.964 (.002)	.966 (.003)
	FiO2	.945 (.006)	.945 (.004)	.943 (.004)
	bolus	.964 (.005)	.965 (.003)	.961 (.004)
	vasopressor	.985 (.002)	.987 (.002)	.988 (.002)
observed-micro	MAP	.997 (.001)	.996 (.0)	.996 (.0)
	DBP	.998 (.0)	.998 (.0)	.998 (.001)
	SBP	.998 (.0)	.998 (.0)	.998 (.0)
	urine output	.961 (.003)	.957 (.004)	.96 (.005)
	ALT	.995 (.001)	.995 (.001)	.995 (.002)
	AST	.996 (.001)	.995 (.001)	.995 (.002)
	pO2	.976 (.002)	.975 (.004)	.973 (.004)
	lactate	.982 (.002)	.981 (.003)	.978 (.004)
	creatinine	.993 (.002)	.993 (.002)	.992 (.003)
	GCS	.965 (.003)	.964 (.004)	.965 (.003)
	FiO2	.944 (.005)	.942 (.006)	.939 (.006)
	bolus	.97 (.004)	.967 (.005)	.969 (.004)
	vasopressor	.986 (.001)	.986 (.001)	.987 (.001)
vanilla	MAP	.996 (.0)	.994 (.0)	.99 (.001)
	DBP	.997 (.0)	.995 (.0)	.991 (.002)
	SBP	.997 (.0)	.995 (.0)	.991 (.002)
	urine output	.981 (.002)	.961 (.003)	.888 (.009)
	ALT	.997 (.001)	.997 (.001)	.991 (.003)
	AST	.998 (.001)	.997 (.001)	.992 (.003)
	pO2	.985 (.001)	.977 (.002)	.958 (.006)
	lactate	.988 (.002)	.977 (.002)	.959 (.006)
	creatinine	.996 (.001)	.992 (.003)	.976 (.005)
	GCS	.995 (.002)	.965 (.002)	.915 (.008)
	FiO2	.979 (.003)	.958 (.004)	.903 (.011)
	bolus	.976 (.005)	.966 (.004)	.956 (.008)
	vasopressor	.992 (.001)	.984 (.001)	.971 (.004)

Balanced accuracy (higher is better) of mask reconstruction on test set per variable. Average and standard deviation across 20 training runs per loss normalization and imputation method. Mask encoding is used for all configurations.

Table S2: Value reconstruction results with encoded mask

Loss normalization	Metric	Variable	MM	MICE-linear	MissForest
observed-macro	MAE	MAP	.195 (.007)	.206 (.008)	.215 (.009)
		DBP	.216 (.008)	.228 (.008)	.235 (.01)
		SBP	.245 (.01)	.252 (.01)	.262 (.014)
		urine output	.295 (.016)	.296 (.012)	.306 (.015)
		ALT	.162 (.011)	.185 (.019)	.207 (.014)
		AST	.159 (.014)	.19 (.017)	.205 (.014)
		pO2	.259 (.004)	.284 (.013)	.297 (.018)
		lactate	.227 (.003)	.248 (.007)	.253 (.019)
		creatinine	.088 (.005)	.162 (.011)	.152 (.023)
	Kendall's tau	GCS	.976 (.002)	.968 (.001)	.966 (.005)
		FiO2	.949 (.007)	.938 (.007)	.925 (.007)
		bolus	.946 (.007)	.937 (.008)	.93 (.008)
		vasopressor	.987 (.001)	.985 (.001)	.985 (.001)
observed-micro	MAE	MAP	.124 (.003)	.124 (.003)	.125 (.003)
		DBP	.152 (.003)	.153 (.004)	.154 (.004)
		SBP	.182 (.007)	.184 (.006)	.188 (.005)
		urine output	.24 (.023)	.251 (.02)	.267 (.02)
		ALT	.404 (.052)	.429 (.056)	.564 (.105)
		AST	.396 (.047)	.416 (.046)	.533 (.096)
		pO2	.466 (.009)	.492 (.009)	.552 (.004)
		lactate	.387 (.018)	.421 (.013)	.486 (.029)
		creatinine	.265 (.012)	.313 (.017)	.316 (.021)
	Kendall's tau	GCS	.976 (.002)	.968 (.004)	.971 (.003)
		FiO2	.943 (.007)	.931 (.007)	.921 (.008)
		bolus	.92 (.01)	.89 (.02)	.881 (.019)
		vasopressor	.993 (.001)	.992 (.001)	.993 (.001)
vanilla	MAE	MAP	.218 (.007)	.282 (.009)	.378 (.027)
		DBP	.243 (.007)	.31 (.008)	.396 (.022)
		SBP	.278 (.012)	.337 (.007)	.414 (.017)
		urine output	.391 (.01)	.398 (.008)	.494 (.021)
		ALT	.801 (.012)	.789 (.018)	.564 (.056)
		AST	.803 (.012)	.75 (.015)	.494 (.072)
		pO2	.644 (.006)	.547 (.009)	.506 (.015)
		lactate	.712 (.014)	.459 (.026)	.502 (.027)
		creatinine	.717 (.039)	.617 (.021)	.57 (.032)
	Kendall's tau	GCS	.96 (.008)	.905 (.003)	.86 (.015)
		FiO2	.873 (.014)	.881 (.01)	.749 (.049)
		bolus	.91 (.012)	.938 (.013)	.976 (.012)
		vasopressor	.976 (.001)	.973 (.003)	.983 (.006)

Test set value reconstruction results for observed data points per variable. Average and standard deviation across 20 training runs per loss normalization and imputation method. Mask encoding is used for all configurations. Performance is measured in mean absolute error (lower is better) for metric variables, and in Kendall's tau rank correlation coefficient (higher is better) for ordinal class variables.

Table S3: Value reconstruction results without encoded mask

Loss normalization	Metric	Variable	MM	MICE-linear	MissForest
observed-macro	MAE	MAP	.127 (.004)	.155 (.005)	.226 (.005)
		DBP	.154 (.005)	.188 (.005)	.264 (.004)
		SBP	.184 (.007)	.234 (.007)	.315 (.004)
		urine output	.201 (.012)	.258 (.007)	.411 (.007)
		ALT	.162 (.007)	.184 (.008)	.545 (.035)
		AST	.16 (.01)	.179 (.009)	.482 (.026)
		pO2	.257 (.016)	.282 (.005)	.402 (.016)
		lactate	.216 (.012)	.25 (.005)	.398 (.02)
		creatinine	.097 (.004)	.181 (.01)	.365 (.016)
	Kendall's tau	GCS	.978 (.001)	.975 (.004)	.93 (.007)
		FiO2	.951 (.007)	.926 (.003)	.802 (.02)
		bolus	.952 (.008)	.921 (.008)	.804 (.015)
		vasopressor	.988 (.001)	.989 (.001)	.973 (.003)
observed-micro	MAE	MAP	.097 (.002)	.099 (.002)	.111 (.006)
		DBP	.106 (.003)	.116 (.003)	.142 (.007)
		SBP	.113 (.005)	.137 (.004)	.18 (.012)
		urine output	.152 (.006)	.196 (.009)	.274 (.036)
		ALT	.392 (.021)	.391 (.02)	.776 (.014)
		AST	.394 (.019)	.382 (.019)	.75 (.018)
		pO2	.404 (.007)	.401 (.006)	.5 (.007)
		lactate	.347 (.007)	.351 (.009)	.542 (.013)
		creatinine	.244 (.004)	.302 (.008)	.596 (.012)
	Kendall's tau	GCS	.978 (.001)	.98 (.007)	.988 (.002)
		FiO2	.939 (.005)	.936 (.004)	.883 (.006)
		bolus	.896 (.006)	.863 (.014)	.371 (.027)
		vasopressor	.996 (.001)	.997 (.0)	.993 (.001)
vanilla	MAE	MAP	.105 (.002)	.139 (.005)	.24 (.025)
		DBP	.113 (.004)	.165 (.005)	.286 (.018)
		SBP	.131 (.005)	.201 (.009)	.334 (.012)
		urine output	.164 (.006)	.209 (.008)	.419 (.037)
		ALT	.813 (.012)	.802 (.016)	.481 (.07)
		AST	.822 (.011)	.766 (.015)	.383 (.076)
		pO2	.664 (.011)	.509 (.011)	.481 (.016)
		lactate	.784 (.007)	.444 (.026)	.457 (.029)
		creatinine	.791 (.013)	.573 (.024)	.498 (.036)
	Kendall's tau	GCS	.98 (.001)	.941 (.004)	.922 (.02)
		FiO2	.962 (.004)	.981 (.003)	.916 (.027)
		bolus	.984 (.003)	.995 (.002)	.995 (.004)
		vasopressor	.994 (.001)	.994 (.001)	.996 (.004)

Test set value reconstruction results for observed data points per variable. Average and standard deviation across 20 training runs per loss normalization and imputation method. Mask encoding is never used. Performance is measured in mean absolute error (lower is better) for metric variables, and in Kendall's tau rank correlation coefficient (higher is better) for ordinal class variables.

Table S4: Downstream survival classification task results

Mask encoded	Loss normalization	Imputation mode	linear	SVM	single layer	MLP
yes	observed-macro	MM	**.858 (.021)**	.841 (.025)	.833 (.022)	.854 (.021)
		MICE-linear	.847 (.021)	**.848 (.024)**	.825 (.023)	.848 (.024)
		MissForest	.852 (.017)	.848 (.026)	**.839 (.017)**	**.858 (.019)**
	observed-micro	MM	.846 (.018)	.829 (.025)	.815 (.021)	.834 (.018)
		MICE-linear	.848 (.018)	.838 (.025)	.819 (.025)	.84 (.022)
		MissForest	.844 (.019)	.838 (.028)	.827 (.021)	.842 (.025)
	vanilla	MM	.837 (.019)	.812 (.026)	.817 (.019)	.839 (.019)
		MICE-linear	.84 (.019)	.833 (.025)	.818 (.023)	.84 (.026)
		MissForest	.825 (.021)	.813 (.025)	.801 (.023)	.822 (.021)
no	observed-macro	MM	.652 (.013)	.652 (.02)	.622 (.016)	.646 (.017)
		MICE-linear	.669 (.018)	.696 (.017)	.626 (.018)	.656 (.018)
		MissForest	.761 (.02)	.744 (.026)	.736 (.024)	.755 (.019)
	observed-micro	MM	.643 (.021)	.659 (.022)	.6 (.014)	.62 (.015)
		MICE-linear	.676 (.021)	.699 (.024)	.632 (.019)	.656 (.022)
		MissForest	.757 (.024)	.749 (.023)	.728 (.021)	.749 (.021)
	vanilla	MM	.605 (.028)	.609 (.021)	.576 (.017)	.593 (.019)
		MICE-linear	.687 (.022)	.705 (.024)	.651 (.023)	.684 (.024)
		MissForest	.8 (.024)	.788 (.027)	.727 (.034)	.77 (.03)

Downstream classification task balanced accuracy (higher is better) for logistic regression (linear), support vector machine (SVM), single-layer perceptron (single layer), and multi-layer perceptron (MLP) models. Average and standard deviation across 20 AE training runs per configuration of mask encoding, loss normalization, and imputation mode. Best results per model are depicted in bold.

Table S5: Downstream length-of-stay regression task results

Mask encoded	Loss normalization	Imputation mode	linear	SVM	single layer	MLP
yes	observed-macro	MM	**2.199 (.074)**	1.874 (.084)	1.984 (.08)	**1.946 (.078)**
		MICE-linear	2.206 (.077)	1.878 (.08)	1.99 (.08)	1.951 (.072)
		MissForest	2.202 (.072)	1.875 (.082)	1.984 (.077)	1.947 (.076)
	observed-micro	MM	2.209 (.077)	1.876 (.085)	1.989 (.082)	1.95 (.079)
		MICE-linear	2.2 (.077)	1.876 (.085)	**1.982 (.082)**	1.949 (.078)
		MissForest	2.202 (.08)	**1.872 (.087)**	1.986 (.085)	1.95 (.082)
	vanilla	MM	2.207 (.077)	1.904 (.086)	1.994 (.086)	1.99 (.08)
		MICE-linear	2.237 (.077)	1.893 (.086)	2.019 (.085)	1.979 (.08)
		MissForest	2.297 (.078)	1.929 (.094)	2.067 (.087)	2.016 (.09)
no	observed-macro	MM	2.753 (.074)	2.072 (.087)	2.556 (.08)	2.372 (.092)
		MICE-linear	2.795 (.088)	2.121 (.09)	2.554 (.085)	2.386 (.091)
		MissForest	2.618 (.09)	2.136 (.099)	2.377 (.098)	2.284 (.098)
	observed-micro	MM	2.78 (.084)	2.077 (.09)	2.602 (.086)	2.424 (.088)
		MICE-linear	2.801 (.086)	2.125 (.088)	2.566 (.084)	2.46 (.096)
		MissForest	2.587 (.079)	2.117 (.094)	2.36 (.09)	2.268 (.092)
	vanilla	MM	2.789 (.082)	2.093 (.088)	2.634 (.087)	2.446 (.089)
		MICE-linear	2.753 (.088)	2.132 (.093)	2.531 (.093)	2.44 (.1)
		MissForest	2.4 (.103)	2.014 (.098)	2.167 (.111)	2.137 (.096)

Downstream regression task mean absolute error (lower is better) for linear regression (linear), support vector machine (SVM), single-layer perceptron (single layer), and multi-layer perceptron (MLP) models. Average and standard deviation across 20 AE training runs per configuration of mask encoding, loss normalization, and imputation mode. Best results per model are depicted in bold.

Table S6: Synthetic variable missing rate deviation

Loss normalization	Variable	MM	MICE-linear	MissForest
observed-macro	MAP	.037 (.008)	.036 (.006)	.033 (.005)
	DBP	.036 (.008)	.035 (.006)	.032 (.005)
	SBP	.035 (.008)	.034 (.006)	.032 (.005)
	urine output	.054 (.017)	.044 (.018)	.052 (.014)
	ALT	.02 (.003)	.02 (.003)	.019 (.002)
	AST	.02 (.003)	.02 (.002)	.019 (.002)
	pO2	.062 (.005)	.063 (.006)	.058 (.005)
	lactate	.089 (.006)	.088 (.006)	.084 (.007)
	creatinine	.14 (.007)	.136 (.007)	.135 (.007)
	GCS	.173 (.032)	.149 (.022)	.152 (.026)
	FiO2	.063 (.008)	.058 (.01)	.061 (.01)
	bolus	.025 (.004)	.026 (.003)	.025 (.002)
	vasopressor	.047 (.006)	.042 (.009)	.042 (.008)
observed-micro	MAP	.037 (.013)	.037 (.007)	.045 (.008)
	DBP	.036 (.012)	.036 (.008)	.044 (.008)
	SBP	.035 (.013)	.035 (.007)	.044 (.008)
	urine output	.066 (.013)	.065 (.015)	.064 (.01)
	ALT	.019 (.008)	.017 (.003)	.02 (.003)
	AST	.019 (.008)	.017 (.003)	.02 (.003)
	pO2	.06 (.012)	.058 (.007)	.063 (.009)
	lactate	.081 (.015)	.081 (.006)	.088 (.01)
	creatinine	.134 (.016)	.131 (.006)	.138 (.007)
	GCS	.168 (.036)	.155 (.018)	.178 (.027)
	FiO2	.063 (.008)	.056 (.01)	.063 (.009)
	bolus	.034 (.008)	.036 (.004)	.039 (.004)
	vasopressor	.049 (.014)	.049 (.008)	.043 (.012)
vanilla	MAP	.038 (.006)	.039 (.005)	.039 (.006)
	DBP	.037 (.006)	.039 (.005)	.038 (.006)
	SBP	.036 (.006)	.037 (.005)	.037 (.006)
	urine output	.073 (.01)	.066 (.014)	.063 (.011)
	ALT	.017 (.002)	.015 (.004)	.014 (.002)
	AST	.017 (.002)	.015 (.004)	.014 (.002)
	pO2	.054 (.005)	.046 (.005)	.044 (.004)
	lactate	.078 (.006)	.071 (.006)	.068 (.006)
	creatinine	.125 (.004)	.131 (.006)	.122 (.004)
	GCS	.102 (.007)	.099 (.007)	.122 (.01)
	FiO2	.051 (.006)	.048 (.006)	.052 (.008)
	bolus	.027 (.002)	.029 (.002)	.034 (.004)
	vasopressor	.03 (.008)	.034 (.008)	.023 (.011)

Absolute difference (lower is better) between synthetic an test set missing rate per variable. Average and standard deviation across 20 training runs per loss normalization and imputation method. Mask encoding is used for all configurations.

Table S7: Synthetic data distribution distances with encoded mask

Loss normalization	Metric	Variable	MM	MICE-linear	MissForest
observed-macro	Wasserstein distance	MAP	.295 (.009)	.296 (.009)	.295 (.014)
		DBP	.295 (.008)	.294 (.011)	.293 (.013)
		SBP	.288 (.008)	.287 (.013)	.286 (.013)
		urine output	.23 (.02)	.242 (.029)	.247 (.017)
		ALT	.127 (.027)	.114 (.031)	.15 (.022)
		AST	.148 (.029)	.131 (.035)	.157 (.022)
		pO2	.285 (.022)	.31 (.021)	.326 (.024)
		lactate	.342 (.029)	.344 (.038)	.349 (.031)
		creatinine	.136 (.023)	.142 (.021)	.139 (.02)
	Mean absolute class ratio difference	GCS	.009 (.002)	.008 (.001)	.008 (.001)
		FiO2	.008 (.002)	.009 (.002)	.009 (.002)
		bolus	.026 (.014)	.033 (.014)	.033 (.009)
		vasopressor	.019 (.008)	.024 (.01)	.024 (.006)
observed-micro	Wasserstein distance	MAP	.23 (.015)	.232 (.008)	.222 (.014)
		DBP	.237 (.019)	.239 (.01)	.23 (.013)
		SBP	.232 (.015)	.233 (.01)	.222 (.014)
		urine output	.248 (.011)	.26 (.014)	.272 (.015)
		ALT	.365 (.032)	.37 (.016)	.479 (.018)
		AST	.353 (.033)	.361 (.018)	.462 (.02)
		pO2	.458 (.018)	.457 (.019)	.47 (.017)
		lactate	.426 (.033)	.422 (.016)	.473 (.019)
		creatinine	.406 (.016)	.427 (.016)	.5 (.015)
	Mean absolute class ratio difference	GCS	.01 (.001)	.009 (.001)	.009 (.001)
		FiO2	.009 (.005)	.01 (.002)	.01 (.002)
		bolus	.07 (.014)	.101 (.018)	.188 (.015)
		vasopressor	.022 (.008)	.019 (.007)	.017 (.007)
vanilla	Wasserstein distance	MAP	.381 (.013)	.383 (.01)	.376 (.019)
		DBP	.375 (.011)	.378 (.012)	.374 (.017)
		SBP	.373 (.011)	.37 (.009)	.369 (.015)
		urine output	.66 (.017)	.498 (.012)	.45 (.018)
		ALT	.763 (.013)	.744 (.017)	.622 (.043)
		AST	.771 (.013)	.712 (.014)	.557 (.043)
		pO2	.708 (.007)	.6 (.009)	.536 (.02)
		lactate	.741 (.008)	.517 (.022)	.521 (.028)
		creatinine	.757 (.01)	.656 (.01)	.566 (.039)
	Mean absolute class ratio difference	GCS	.037 (.002)	.025 (.001)	.02 (.004)
		FiO2	.039 (.003)	.019 (.003)	.094 (.009)
		bolus	.142 (.014)	.111 (.012)	.115 (.03)
		vasopressor	.042 (.006)	.054 (.007)	.035 (.012)

Distance metrics between synthetic data and test set per variable. Average and standard deviation across 20 training runs per loss normalization and imputation method. Mask encoding is used for all configurations. Distance is measured as Wasserstein distance (lower is better) for metric variables and mean absolute difference in class ratios (lower is better) for ordinal class variables.

Table S8: Synthetic data distribution distances without encoded mask

Loss normalization	Metric	Variable	MM	MICE-linear	MissForest
observed-macro	Wasserstein distance	MAP	.322 (.007)	.318 (.008)	.315 (.007)
		DBP	.32 (.004)	.32 (.009)	.309 (.007)
		SBP	.316 (.01)	.312 (.01)	.307 (.01)
		urine output	.314 (.012)	.305 (.012)	.305 (.01)
		ALT	.389 (.026)	.369 (.024)	.486 (.021)
		AST	.399 (.023)	.38 (.024)	.468 (.022)
		pO2	.474 (.01)	.505 (.013)	.614 (.012)
		lactate	.486 (.018)	.516 (.019)	.665 (.02)
		creatinine	.177 (.016)	.206 (.021)	.399 (.017)
	Mean absolute class ratio difference	GCS	.013 (.002)	.01 (.002)	.008 (.001)
		FiO2	.035 (.002)	.041 (.002)	.04 (.002)
		bolus	.322 (.004)	.282 (.011)	.196 (.012)
		vasopressor	.1 (.008)	.113 (.007)	.132 (.009)
observed-micro	Wasserstein distance	MAP	.258 (.009)	.256 (.007)	.254 (.009)
		DBP	.264 (.01)	.26 (.008)	.253 (.01)
		SBP	.254 (.01)	.253 (.005)	.242 (.008)
		urine output	.335 (.011)	.326 (.012)	.326 (.013)
		ALT	.496 (.019)	.51 (.032)	.65 (.017)
		AST	.484 (.016)	.481 (.03)	.622 (.014)
		pO2	.59 (.014)	.58 (.016)	.63 (.018)
		lactate	.553 (.019)	.511 (.023)	.595 (.027)
		creatinine	.438 (.015)	.473 (.023)	.55 (.013)
	Mean absolute class ratio difference	GCS	.014 (.001)	.011 (.002)	.009 (.002)
		FiO2	.031 (.002)	.038 (.002)	.039 (.003)
		bolus	.207 (.008)	.166 (.009)	.294 (.01)
		vasopressor	.177 (.004)	.11 (.007)	.109 (.013)
vanilla	Wasserstein distance	MAP	.422 (.012)	.416 (.013)	.373 (.051)
		DBP	.424 (.01)	.417 (.012)	.373 (.045)
		SBP	.418 (.015)	.408 (.012)	.375 (.047)
		urine output	.71 (.008)	.572 (.012)	.461 (.046)
		ALT	.806 (.012)	.798 (.013)	.679 (.083)
		AST	.816 (.012)	.778 (.012)	.602 (.075)
		pO2	.773 (.006)	.714 (.006)	.638 (.04)
		lactate	.787 (.007)	.666 (.01)	.654 (.059)
		creatinine	.79 (.007)	.693 (.015)	.533 (.076)
	Mean absolute class ratio difference	GCS	.056 (.004)	.054 (.006)	.018 (.006)
		FiO2	.119 (.005)	.047 (.005)	.095 (.01)
		bolus	.361 (.004)	.303 (.013)	.129 (.047)
		vasopressor	.363 (.007)	.22 (.01)	.093 (.012)

Distance metrics between synthetic data and test set per variable. Average and standard deviation across 20 training runs per loss normalization and imputation method. Mask encoding is never used. Distance is measured as Wasserstein distance (lower is better) for metric variables and mean absolute difference in class ratios (lower is better) for ordinal class variables.
