# Supplementary material for: MANNERS: A strategy for representation learning in multivariate datasets with high proportions of missing data
Source: Patterns (N Y). 2026 Apr 23;7(7):101543. doi: 10.1016/j.patter.2026.101543 (PMC13366519; doi:10.1016/j.patter.2026.101543)
Supplement: Document S2. Article plus supplemental information [file mmc2.pdf]

Article

Patterns

MANNERS: A strategy for representation learning in multivariate datasets with high proportions of missing data

Highlights

- Learns from missing data instead of relying solely on imputation
- Handles 99% missing rates in multivariate EHR time series
- Encodes missingness, masks loss, and rebalances sparse signals
- Improves reconstruction, prediction, and data generation tasks

Authors

Louis Bellmann, Maximilian Nielsen, Philipp Breitfeld

Correspondence

l.bellmann@uke.de

In brief

Missing data are a major challenge in real-world datasets, especially in healthcare, where measurements are often incomplete by design. Most methods attempt to fill in these gaps, but important information can be lost in the process. Bellmann et al. show that missing data themselves can be informative and propose a learning strategy for representation learning that leverages these patterns. By shifting from imputing missing values to learning from them, their approach improves model performance in highly sparse settings.

Bellmann et al., 2026, Patterns 7, 101543
July 10, 2026 © 2026 The Authors. Published by Elsevier Inc.
https://doi.org/10.1016/j.patter.2026.101543

CellPress

Patterns

CellPress
OPEN ACCESS

Article

MANNERS: A strategy for representation learning in multivariate datasets with high proportions of missing data

Louis Bellmann,[1,2,*] Maximilian Nielsen,[1] and Philipp Breitfeld[1]
[1]Institute for Applied Medical Informatics, University Medical Center Hamburg-Eppendorf, Hamburg, Germany
[2]Lead contact
*Correspondence: l.bellmann@uke.de
https://doi.org/10.1016/j.patter.2026.101543

THE BIGGER PICTURE Today, deep learning shapes the scientific world. In representation learning, a model extracts compact embeddings from complex input data, capturing data patterns to enable downstream tasks. Missing parts of the input data must be filled for the deep learning model to function. Large quantities of missing data are a major problem in representation learning since embedding quality, and therefore downstream task performance, is reduced. Medical time-resolved data from electronic health records often exhibit very large missing data rates. This reflects clinical practice, as some tests are performed only when medically indicated, while others are measured at differing intervals. In this work, we propose a training strategy for representation learning that accounts for missing data and treats them as a source of information. Our approach improves performance in three application settings, and we show that the learned embeddings encode clinically relevant information stemming from missing data.

SUMMARY

Missing data present a major challenge for deep learning, and various imputation techniques exist. However, imputation quality generally decreases as missing data rates increase. In time-series data from electronic health records, missing rates for individual parameters up to 99% can be observed, posing a problem for mere imputation. In addition, underlying patterns between missing and observed data can contain valuable information that can be learned by deep learning models. In this work, we propose a strategy called missing adjusted normalization and nullity encoding representation strategy (MANNERS), which can be applied to training pipelines for representation learning. MANNERS encodes missingness, masks loss evaluation at missing data points, and applies rebalancing such that the signal from variables with high missing rates is not lost. We evaluated MANNERS on reconstruction and downstream classification, regression, and synthetic data generation tasks. We showed performance improvements in the presence of very high missing rates compared with state-of-the-art imputation-only techniques.

INTRODUCTION

Missing data occur frequently in real-world datasets across many domains and pose a challenge for data analysis and machine learning applications.[1–5] In time series from electronic health records (EHRs), missing rates, i.e., the fraction of data that is missing per variable, can reach up to 99%.[6,7] This phenomenon is partially caused by varying measurement frequencies of laboratory and medical tests, many of which are conducted only when clinically indicated (e.g., in response to worsening patient conditions). When time-resolved data are quantized, e.g., to hourly values, this difference in measurement frequencies can lead to large missing rates, as infrequent measurement types must be set as missing for many time steps.[8,9] These extremely large missing rates present problems[8,10–12] for the growing toolbox of machine and deep learning techniques on time series, such as time-series decomposition,[13] wavelet transforms,[14–16] contrastive learning,[17,18] masked autoencoders (AEs),[19–21] and auto-correlation-based transformers.[22,23]

In the state of the art, missing data are imputed effectively, filling the missing parts of the dataset, and a plethora of technologies exist.[4,10] The methods range from simply filling with a fixed value and using traditional machine learning techniques, such as multiple imputation by chained equations (MICE)[24,25] and MissForest,[26] to employing deep learning techniques that use

This is an open access article under the CC BY license (http://creativecommons.org/licenses/by/4.0/).

generative adversarial networks,[27,28] denoising AEs,[29,30] and masked AEs.[19,20] Regardless of the imputation method employed, these now artificially completed datasets are used for the desired application as the next independent step. This approach comes with two potential problems when faced with extremely large missing rates. Firstly, imputation errors generally increase with increasing missing rates.[31–34] Secondly, if missing data are not missing by chance, underlying patterns between missing and observed data might exist, and this information is lost by relying solely on imputation and resulting artificially completed datasets. After all, the motivation of imputation is to make imputed missing data points indistinguishable from observed data points. Removing these patterns can be harmful for downstream applications.[8,35]

Several studies have reported a reduction in imputation quality across imputation techniques as the proportion of missing data increases.[31–34] This trend is intuitive, as higher missing rates leave less observed data to derive the missing data from. This decrease in imputation performance constitutes a problem for time-series data from EHRs with the potential missing rates of 99% described above. Additionally, several studies found that the more complex deep learning-based imputation methods do not necessarily yield better imputation performance than simpler traditional machine learning methods.[33–36]

Regarding the second point, missing data can be categorized by the underlying reason for their missingness, following Rubin et al.[37]: when the missingness of a variable depends on other observed variables in a dataset, the missing data are called missing at random (MAR). In contrast, if missingness is independent of both observed and unobserved data, it is called missing completely at random (MCAR). State-of-the-art imputation techniques, such as MICE[24] and MissForest,[26] estimate missing values using relationships between observed variables. For statistical validity, these methods typically rely on the assumption that the missingness mechanism is MAR (or the stronger MCAR assumption).

Turning this relationship around, missing data can also contain information about the observed data: e.g., a missing laboratory measurement might indicate that a patient's observed vital signs were viewed as uncritical by the hospital staff, a missing medical treatment could mean the stabilization of a patient's health status. Such informative relationships between missingness patterns and observed variables have been shown to be useful for downstream machine learning tasks.[8,35] When datasets are made complete through imputation alone, these potentially informative missingness patterns may be obscured or lost.

For these reasons, we explore a new approach to handling large missing rates of 90% and above in multivariate time-series data by leveraging representation learning and encoding missing data patterns directly into the learned representations. The proposed strategy moves beyond conventional preprocessing via imputation to address missing data. Instead, we modify the training pipeline for reconstruction-based representation learning to explicitly account for missingness. The learned representations can then be used to solve the desired task without the need for additional handling of missing data, while the underlying informative missing data patterns can be leveraged. The missing adjusted normalization and nullity encoding representation strategy (MANNERS) adds the following steps to a reconstruction task.

(1) Generate a binary missing mask indicating whether each data point was originally observed or missing. Encode the missing mask into the representation by adding it to the reconstruction task.
(2) Exclude missing data points from the elementwise value reconstruction loss, leveraging the missing mask.
(3) Apply macro averaging to the adjusted elementwise value reconstruction loss, i.e., average the elementwise loss for each variable individually and then average the result across variables.

Steps 1 and 2 have already been part of investigations: a binary missing mask was already leveraged by others for missing data imputation,[38,39] synthetic incomplete data generation,[40] or as input for classic machine learning classification models.[35] Exclusion of missing data points from loss calculations was employed by others for missing data imputation.[24,41] However, the main contributions of this work are the summarization of these ideas, the addition of the normalization strategy in step 3, and the direct application of the summarized steps to representation learning independent of missing data imputation. To evaluate the impact of the proposed strategy, we analyze the performance of the learned representation in downstream, reconstruction, and synthetic data generation tasks. Moreover, we thoroughly investigate the impact of the individual steps 1–3 of MANNERS on said performance in an ablation study.

For evaluation, we use a multivariate time-series dataset derived from EHRs that exhibits missing rates of up to 98% and contains informative patterns between observed and missing data. Although our experiments focus on this setting, the proposed MANNERS approach is, in principle, also applicable to univariate and non-temporal data with high levels of missingness. We train AEs with full MANNERS but also exclude individual steps and investigate the state-of-the-art approach of sole imputation. Further, we combine the different training configurations with three common imputation techniques: imputation by mean/median (MM) value, MICE,[24] and MissForest.[26] Thereby, we analyze the impact of imputation methods with varying complexity on the training configurations.

We show that AEs with MANNERS achieve significantly better results in surrogate test set reconstruction for variables with high missing rates, as well as modestly improved performance in downstream classification and regression tasks. Moreover, variational AEs (VAEs) trained with MANNERS show a significantly improved goodness of fit between original and synthetic data when high proportions of missing data are present.

RESULTS

In three separate experiments, we investigated the impact of different missing data imputation methods and the proposed MANNERS methodology on model performance in the presence of moderate-to-high (above 90%) variable missing rates on tasks commonly associated with representation learning. To this end, we employed a standard fully convolutional AE architecture comprising three encoder and three decoder layers.[42] As data modality, we chose fixed-length, multivariate time series where each input is a $d \times t$ matrix with d variables and t time steps. Each input matrix contains at least one missing data point.

CellPress
OPEN ACCESS

AEs process input matrices as a whole to generate one representation for each multivariate time series.

Missing data were imputed using three techniques: with the MM method, missing data points are filled with a fixed value given by the mean for metric variables and the median for categorical variables. The MICE-linear method utilized MICE[24] together with Bayesian ridge regression,[43] which has been shown to outperform deep learning-based imputation in several studies.[44,45] Finally, we use the MissForest imputation method, combining the MICE approach with random forest models.[26] All three imputation methods were fitted only using the AE training data and applied to training, validation, and test data.

For the ablation study, AEs were trained with the same architecture but different training configurations that incorporate none, some, or all steps of MANNERS. With all configurations, a surrogate reconstruction task for metric and ordinal class variables is solved. If the mask encoding of MANNERS step 1 is used in the configuration, a binary mask reconstruction is added to the overall reconstruction task. In vanilla loss normalization, loss is not masked for missing data points, effectively not using steps 2 and 3 of MANNERS. Since all variables contribute equally as many elements to the vanilla loss normalization, macro and micro averaging yield the same result. In observed-micro loss normalization, the loss is masked for missing data points following step 2 of MANNERS but is then micro-averaged, i.e., directly averaged across all observed data points. Finally, with observed-macro loss normalization, steps 2 and 3 of MANNERS are used. Combining the three imputation techniques, two mask encodings, and three loss normalization strategies, we arrived at 18 different training configurations. To ensure robust results, Monte Carlo cross-validation was employed with 20 randomized train/validation/test splits with time series kept intact and belonging to one of the three sets. As all individual time series contain missing data, all sets are incomplete. By linking data splits to training configurations, we trained 360 AEs and 360 VAEs, which we compared in three different experiments. Results for all 18 training configurations and all experiments are given in detail in the supplemental information. For clarity, only a subset will be shown in the figures and tables of the manuscript. Results for the remaining configurations will be discussed in the text and exhibit a similar trend. We will always investigate vanilla loss normalization with mask encoding and MM, as well as MissForest imputation, as baselines with the least and the most complex imputation techniques. Besides the configuration with full MANNERS, we also incorporate observed-micro loss normalization for ablation of step 3 in reconstruction and synthesis experiments and omit mask encoding for ablation of step 1 for downstream task performance analysis.

In the first experiment, we assessed the reconstruction quality on a held-out test set. Low reconstruction error is particularly important for downstream applications that rely on the fidelity of the AE's latent space, such as latent-space diffusion models,[46] where the quality of generated samples depends heavily on the quality of the learned representations. In the second experiment, downstream performance was evaluated on classification and regression tasks using the learned and fixed latent representations as input features for four different downstream models. In the third experiment, we applied the same 18 training configurations to VAEs, where the latent space is modeled as a Gaussian distribution, as is standard in VAE architectures.[47] In this case, the quality of the synthetic data generated by the model was assessed based on their fit on the test set and preservation of inter-variable correlations.

For our experiments, we chose a publicly available multivariate time-series dataset defined by Gottesman et al.[48] considering the intensive care unit (ICU) management of acutely hypotensive patients within the first 48 h. The dataset was extracted from the MIMIC-IV database[49] and contains 13 hourly time-resolved variables, of which three are mean, diastolic, and systolic arterial blood pressure (MAP, DBP, and SBP, respectively). Additionally, urine output, Glasgow Coma Scale (GCS)[50] evaluations, and the laboratory measurements serum creatinine, lactate, partial pressure of oxygen (pO_2), aspartate aminotransferase (AST), and alanine aminotransferase (ALT). Finally, 3 types of treatments were reported: the fraction of inspired oxygen used for ventilation (FiO_2), vasopressor infusion, and administered fluid boluses. We applied padding with missing values when patients stayed for less than 48 h and added a missing mask for all variables. This dataset was chosen for two reasons: firstly, the variables of the dataset exhibit moderate to very high variable missing rates. Therefore, the dataset is well suited for analyzing the influence of missing rates on the training setups to compare. Secondly, the administration of medical treatments and the ordering of laboratory measurements depend on the patient's health status, e.g., blood pressure and GCS. Therefore, a MAR characteristic following the definition of Rubin et al.[37] is present, and patterns between observed and missing data exist.

The novel MANNERS methodology, model architecture, training setup, and dataset definitions, as well as preprocessing, are discussed in more detail in the methods section.

Before discussing the three described experiments, we want to quickly point out the run times required by the three imputation methods. In Figure S1, we see that MM imputation requires roughly 0.6 s, MICE-linear 50 s, and MissForest over 3,000 s, exhibiting an almost 100-fold run-time increase between methods.

MANNERS improves data reconstruction with high missing rates

First, we evaluated the test set reconstruction quality of AEs and the impact of the different training configurations. Although data reconstruction from the condensed latent space is only a surrogate task, good reconstruction quality is fundamental for combining AEs with more complex architectures, such as latent diffusion models,[46] or for applying AEs to denoising[51] and anomaly detection[52] tasks. We analyzed value as well as missing mask test set reconstruction quality, and the results are detailed in Tables S1–S3. For value reconstruction quality, we only assessed the originally observed values. When comparing the results for training configurations with mask encoding in Table S2 and configurations without in Table S3, we see that configurations without mask encoding perform better than their counterpart for every configuration. However, configurations without the mask encoding of MANNERS step 1 are not able to reconstruct the missing mask, i.e., generate incomplete data. For this reason, they are not suited for this application, and we only report their results for completeness. From

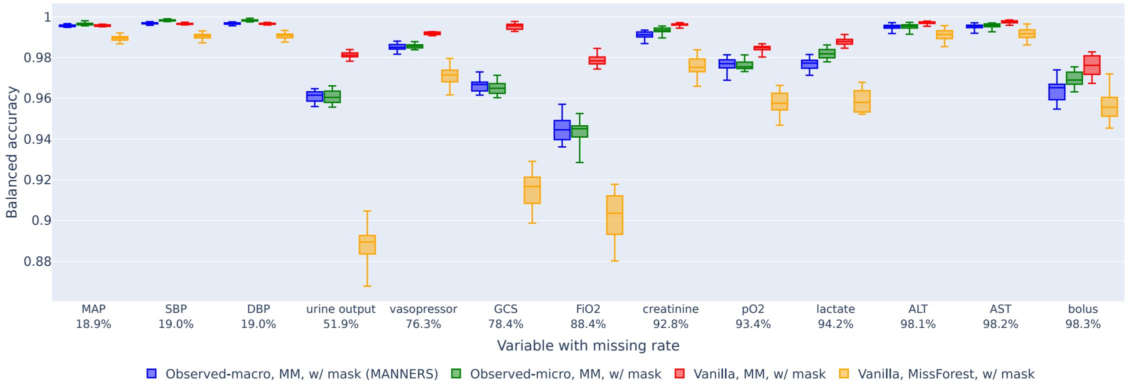

Figure 1. Missing mask reconstruction

Balanced accuracy for missing mask reconstruction on test set per variable across 20 AE training runs for four training configurations with mask encoding: observed-macro loss normalization and MM (mean/median) imputation (MANNERS) (blue), observed-micro loss normalization and MM imputation (green), vanilla loss normalization with MM imputation (red), and loss normalization with MissForest imputation (yellow). The box represents the interquartile range (IQR), the line inside the box indicates the median, and the whiskers extend to the most extreme data points. The missing rate across the whole dataset is given per variable, and variables are sorted by ascending missing rate. Higher is better.

Table S2, we can derive that the employed imputation technique has little effect on the reconstruction results for observed-macro and observed-micro loss normalization using MANNERS step 2. In contrast, reconstruction results differ heavily between imputation methods for vanilla loss normalization. For clarity, we will only compare four different training configurations in the remainder of this subsection: the configuration incorporating the full MANNERS approach with MM imputation, observed-micro loss normalization and mask encoding together with MM imputation, and vanilla loss normalization and mask encoding with both MM and MissForest imputation. The results for the other training configurations follow a similar trend and are reported in Tables S1–S3.

Figure 1 shows the results of reconstructing the missing mask on the test, i.e., the model's ability to learn and reproduce the original patterns of missingness. With a median balanced accuracy of 0.88 and higher, all four configurations reconstruct the missing mask well, and mask reconstruction quality does not depend on variable missing rates despite the class imbalance imposed by high missing rates. In fact, balanced accuracy is highest for MAP, SBP, and DBP, which have the lowest missing rates, and ALT and AST, which have among the highest missing rates. Interestingly, AEs with vanilla loss normalization and MM imputation perform better than the three other configurations for most variables, whereas AEs with vanilla loss normalization and MissForest imputation perform worse and with the highest variability.

In Figure 2, test set value reconstruction results are depicted. Both loss-masking configurations outperform both vanilla loss normalization configurations for all variables except bolus. Overall, AEs trained with full MANNERS show the best reconstruction results for variables with missing rates above 90%, and their variability is the smallest. However, the micro averaging of the observed-micro loss normalization benefits the reconstruction quality of variables with medium missing rates, and this configuration performs best for the remainder of the variables. Regarding the comparison between imputation techniques for vanilla loss normalization, results with MissForest imputation perform better for variables with missing rates above 90% and worse for variables with missing rates below 90%. For AEs with vanilla and observed-micro loss normalization, we can observe a trend of increasing errors with an increase in missing rates, where vanilla loss normalization with MM imputation yields results not much better than randomness for ALT and AST variables. This relationship is not present for AEs trained with MANNERS, which have roughly the same reconstruction errors for all metric variables. Regarding the ordinal class variables, the decrease in Kendall's tau coefficients is smallest in AEs with MANNERS, except for bolus. Moreover, AEs trained with MANNERS show the overall smallest deviations between data splits and thus the highest robustness.

Latent spaces are more informative with MANNERS and yield better downstream task results

In the second experiment, we evaluated the "meaningfulness" of the trained AE latent representations, i.e., how well the representation captures relevant information. To this end, we performed two downstream tasks and analyzed uniform manifold approximation and projections (UMAPs)[54] into $\mathbb{R}^2$ to visually assess the models' ability to structure and disentangle latent features according to clinically relevant patient characteristics. To this end, the length of each ICU stay was extracted from the MIMIC-IV database as fractions of days as a downstream regression task. Additionally, we considered patient survival for the first 48 h of their stay as a classification downstream task. For the analysis, all 18 training configurations were compared. For each configuration and cross-validation split, the trained AE was frozen, and the fixed latent representations were used as input for four different types of downstream models: linear and logistic regression models, support vector machines (SVMs), single-layer perceptrons (SLPs), and multi-layer perceptrons of two fully connected layers connected by Gaussian Error Linear Unit (GELU) activation.[55] Fitting of downstream models was carried out on each split training set and evaluated on the test set. The results can be found in Tables S4 and S5. For clarity, we show results only for the full

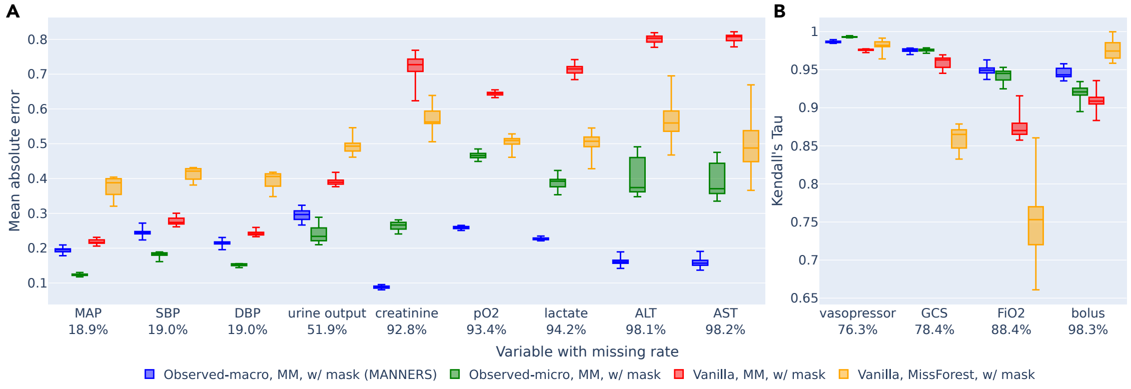

Figure 2. Value reconstruction

Test set value reconstruction results for observed data points per variable across 20 AE training runs for four training configurations with mask encoding: observed-macro loss normalization and MM (mean/median) imputation (MANNERS) (blue), observed-micro loss normalization and MM imputation (green), vanilla loss normalization with MM imputation (red), and loss normalization with MissForest imputation (yellow). The box represents the interquartile range (IQR), the line inside the box indicates the median, and the whiskers extend to the most extreme data points. The missing rate across the whole dataset is given per variable, and variables are sorted by ascending missing rate.

(A) Mean absolute error per metric variable; lower is better.

(B) Kendall's tau rank correlation coefficient[53] for ordinal class variables; higher is better.

MANNERS configuration, the configuration omitting the mask encoding from MANNERS step 1, and all configurations with vanilla loss normalization in Table 1. Additionally, we show results only for SLP and SVM downstream models, as the other two model types and the remaining AE training configurations follow a similar trend. Note that all AEs were only trained for their surrogate reconstruction task without the consideration of survival or length of stay data. To calculate UMAPs, we selected AEs trained on the first cross-validation split for three configurations: full MANNERS, vanilla loss normalization with mask encoding and MM imputation, and vanilla loss normalization with MissForest imputation and without mask encoding. Test set latent representations were projected into $\mathbb{R}^2$, and the results are shown in Figures 3 and S2. The projections were colored by length of stay, survival, and vasopressor missing rate per ICU stay.

In Table 1, we see that AEs trained with MANNERS and simple MM imputation exhibit better results than all configurations with vanilla loss normalization. Tables S4 and S5 show that full MANNERS configurations yield the best results for both tasks and all models except for SLP and SVM downstream model types on the regression task, where observed-micro loss normalization, i.e., omitting MANNERS step 3, achieves the best results. All configurations with mask encoding and observed-macro or observed-micro loss normalization, i.e., employing MANNERS steps 1 and 2, yield the best results for all downstream models and both tasks. However, the mask encoding MANNERS step 1 has the largest impact on downstream task performance, as almost all configurations with mask encoding perform better than all configurations without. Among the configurations with vanilla loss normalization, the most complex MissForest imputation method achieves the best results when the missing mask is not encoded. In stark contrast, vanilla loss normalization and MissForest imputation achieve, on average, the worst results among the configurations with mask encoding. This aligns with the findings of Paterakis et al.,[35] who found that missing mask encoding improves predictive performance when paired with simple imputation methods.

Figure 3 shows UMAPs of representations extracted from the test set using an AE trained with MANNERS and vanilla loss normalization with MissForest imputation as the best-performing configuration without mask encoding. The first row shows the length of stay for individual patients. MANNERS generates much better visual clusters and more even transitions from left to right. For patient survival depicted in the middle row, MANNERS again exhibits much better clustering of non-survivors in the top left and bottom right, with almost no non-survivors in the middle of the plot. In contrast, for vanilla loss normalization with MissForest, non-survivors are much more spread out across the projection plot. These differences directly inform the downstream task performance increase of MANNERS compared with configurations without mask encoding. In Figure S2, we see that a similar comparison can be made between MANNERS and vanilla loss normalization with MM imputation and mask encoding omitting MANNERS steps 2 and 3. However, the differences are smaller, leading to a smaller performance increase. Circling back to the third row of Figure 3, we see that MANNERS generates three well-pronounced clusters with a vasopressor missing rate close to zero, i.e., patients who received vasopressor infusion throughout their whole stay. When we add information from the other two rows, we see that two (top left and bottom right) of these clusters contain many no-survivors, either having very short (top left) or very long (bottom right) ICU stays. In contrast, patients in the middle of the plot have very high missing rates, i.e., receive almost no vasopressor treatments and are mostly survivors with a medium-length stay. This correlation of vasopressor missing rate, survival, and length of stay makes sense from a clinical point of view and qualitatively shows that missing data patterns contain information as discussed by Tan et al.[8] Together with the quantitative results shown in Tables 1, S4, and S5, we see that AEs trained with MANNERS are able to increase downstream task

Table 1. Downstream classification and regression task results

	Survival classification		Length-of-stay regression	
Configuration	SLP	SVM	SLP	SVM
Observed-macro, MM, w/mask (MANNERS)	**0.833 (0.022)**	**0.841 (0.025)**	**1.984 (0.08)**	**1.874 (0.084)**
Vanilla, MM, w/mask	0.817 (0.019)	0.812 (0.026)	1.994 (0.086)	1.904 (0.086)
Vanilla, MICE-linear, w/mask	0.818 (0.023)	0.833 (0.025)	2.019 (0.085)	1.893 (0.086)
Vanilla, MissForest, w/mask	0.801 (0.023)	0.813 (0.025)	2.067 (0.087)	1.929 (0.094)
Observed-macro, MM, w/o mask	0.622 (0.016)	0.652 (0.02)	2.556 (0.08)	2.072 (0.087)
Vanilla, MM, w/o mask	0.576 (0.017)	0.609 (0.021)	2.634 (0.087)	2.093 (0.088)
Vanilla, MICE-linear, w/o mask	0.651 (0.023)	0.705 (0.024)	2.531 (0.093)	2.132 (0.093)
Vanilla, MissForest, w/o mask	0.727 (0.034)	0.788 (0.027)	2.167 (0.111)	2.014 (0.098)

Downstream task results for single-layer perceptron (SLP) and support vector machine (SVM) classification and regression models. Average and standard deviation for 20 AE training runs per training configuration measured in balanced accuracy (higher is better) for the survival classification task and in absolute error (lower is better) for the length-of-stay regression task. The best results are depicted in bold.

performance by capturing these patterns between observed and missing data.

MANNERS improves synthetic data quality

In the last experiment, we analyzed the quality of synthetic data generated by VAEs using the 18 different training configurations. With each of the trained VAEs, we generated a synthetic dataset of the same size as the test set. Note that only configurations with the mask encoding of MANNERS step 1 can generate incomplete synthetic data. For these configurations, we computed the difference in variable-level missing rates, and the results are given in Table S6. Additionally, we evaluated distributional similarities between the observed data points in the synthetic and test sets for all configurations, and the results are documented in Tables S7 and S8. For metric variables, we compared the variable distributions using the Wasserstein distance.[56] For ordinal variables, we calculated the average absolute difference in class fractions.

When comparing the results in Tables S7 and S8, we see that all configurations with mask encoding generate synthetic data of higher fidelity than their counterparts without mask encoding. Paired with the fact that we want to generate incomplete data preserving missing data patterns, we will focus on configurations with mask encoding for the rest of this section. Additionally, we can see that the imputation technique has little effect on synthetic data fidelity when using the loss masking of MANNERS step 2 in observed-macro and observed-micro loss normalization. Similar to the data reconstruction experiment, we focus on four different configurations for clarity: the configuration incorporating the full MANNERS approach with MM imputation, observed-micro loss normalization and mask encoding together with MM imputation, and vanilla loss normalization and mask encoding with both MM and MissForest imputation. The results for the other configurations follow a similar trend.

Additionally, we investigated intra-dataset correlation deviations. To this end, we merged the 20 synthetic datasets and calculated their corresponding Pearson correlation coefficient[57] matrices for each of the nine training configurations with mask encoding. We then subtracted the original correlation coefficients from the synthetic ones to obtain signed correlation deviations, highlighting how well each method preserved inter-variable relationships.

In Figure 4, the deviation of synthetic missing rates from the test sets is shown. For MAP, SBP, DBP, ALT, AST, and bolus, the absolute differences are quite low and relatively similar for all four configurations. For urine output, VAEs trained with MANNERS and MM imputation exhibit the smallest differences. For vasopressor, GCS, FiO_2, creatinine, pO_2, and lactate, one configuration with vanilla loss masking performs best for synthetic mask generation, albeit with a small difference between MM and MissForest imputation. Especially for the GCS variable, the performance of both vanilla configurations is better than that of both configurations with loss masking. Notably, the variability is larger for the observed-micro loss normalization compared with the other configurations. Similar to the findings on missing mask reconstruction, we see that synthetic missing rate fidelity does not depend on variable missing rates despite the imposed class imbalance of missing rates above 90%.

Figure 5 illustrates the differences between the variable distributions of synthetic and test sets. We see that VAEs trained with MANNERS exhibit the lowest distribution discrepancies for all variables except the blood pressure variables MAP, SBP, and DBP, where observed-micro loss normalization performs best. Notably, for configurations with MANNERS, variable distribution differences do not depend on the variable missing rate. In fact, we observe the lowest Wasserstein distance[56] for ALT and AST with the highest missing rate. In contrast, Wasserstein distances for both vanilla loss normalization configurations increase with higher variable missing rates, resulting in a large performance decrease compared with configurations with loss masking. For ordinal class variables, we observe a similar trend. The two vanilla loss normalization configurations are compared, VAEs with MissForest imputation show similar or better results for most variables except FiO_2. For VAEs trained with MANNERS, the fidelity variability between cross-validation data splits is smallest.

In Figure 6, correlation differences between synthetic datasets generated with nine different training configurations and the original dataset are shown. In Figure S3, the same results are depicted with variable names and difference values. Configurations with observed-macro loss normalization preserve the original correlations with the highest fidelity. In contrast, vanilla loss normalization

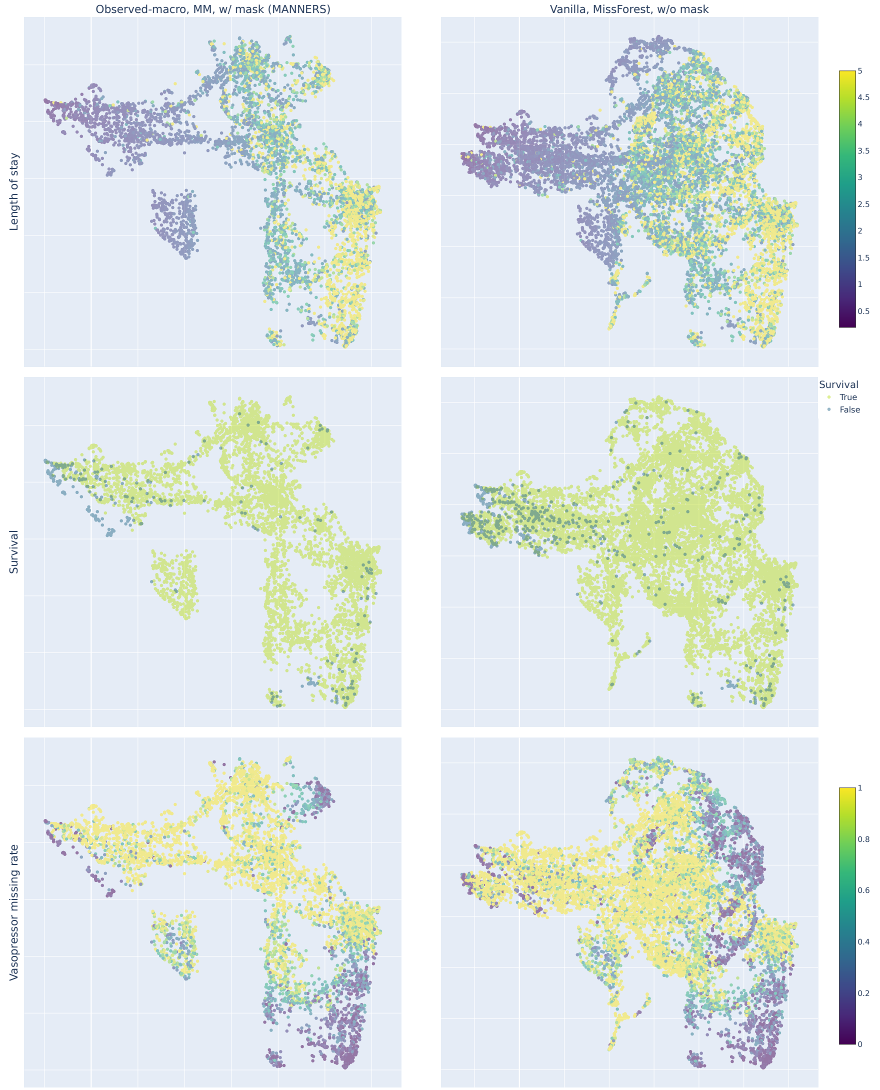

Figure 3. UMAPs of latent representations
Test set representation projections into $\mathbb{R}^2$ on the first Monte Carlo cross-validation split for the configuration with observed-macro loss normalization, MM (mean/median) imputation and mask encoding (MANNERS) (left), and vanilla loss normalization and MissForest imputation without mask encoding (right). Projections are colored by length of ICU stay in fraction of days (top), survival during the first 48 h (middle), and vasopressor missing rate (bottom).

configurations exhibit the greatest deviations from the original correlations and thus the lowest fidelity. For the full MANNERS configuration with MM imputation, we observe low deviations except for the correlation of bolus with ALT and AST, which increases. With this exception, correlation fidelity of configurations with the loss masking of MANNERS step 2, i.e., observed-macro and observed-micro, is largely independent of the chosen imputation method. In contrast, the more advanced MICE-linear and MissForest imputation methods even increase the correlation differences for vanilla loss normalization configurations. This aligns with the findings about variable distribution deviations.

DISCUSSION

In this work, we investigated how to improve representation learning when very high missing rates (above 90%) are present in multivariate datasets. We aimed to overcome the impact of increased imputation error at very high missing rates found by several studies.[31–34] Additionally, we sought to encode underlying patterns between missing and observed data into the learned representations, which are informative for downstream tasks.[8,35] To this end, we introduced MANNERS, a novel training strategy for reconstruction-based representation learning with the following steps: (1) encoding of a binary missing mask into the representation by adding it to the reconstruction task, (2) excluding the missing data points from the elementwise value reconstruction loss, and (3) macro averaging this adjusted elementwise loss by first averaging across variables individually and then averaging the results into the overall loss.

We evaluated the novel methodology on a multivariate time-series dataset from EHRs with missing rates of up to 98% by generating 20 data splits in a Monte Carlo cross-validation and training fully convolutional (V)AEs via a reconstruction task. We compared the MANNERS strategy to the state-of-the-art method of only employing missing data imputation. Additionally, we evaluated the contribution of the individual MANNERS steps in an ablation study. To analyze the impact of imputation methods, we used the MM method of filling missing data with the variable MM, MICE paired with Bayesian ridge regression, and MissForest utilizing random forest models. We benchmarked all configurations on three separate tasks common to representation learning.

In the first experiment, we analyzed test set reconstruction quality and showed that AEs trained with MANNERS significantly outperform vanilla AEs without loss masking in value reconstruction, independent of the imputation technique. This underlines the importance of the loss masking of MANNERS step 2. Observed-micro loss normalization reconstruction error also outperformed vanilla configurations but fell behind full MANNERS configurations with growing variable missing rates. Therefore, we can see that the macro averaging of MANNERS step 3 is crucial for reconstruction variables with missing rates of 90% and above. At lower missing rates, micro averaging, which involves using only MANNERS steps 1 and 2, may also be a feasible approach. For vanilla configurations, MissForest and MICE imputation could not outperform MM imputation,

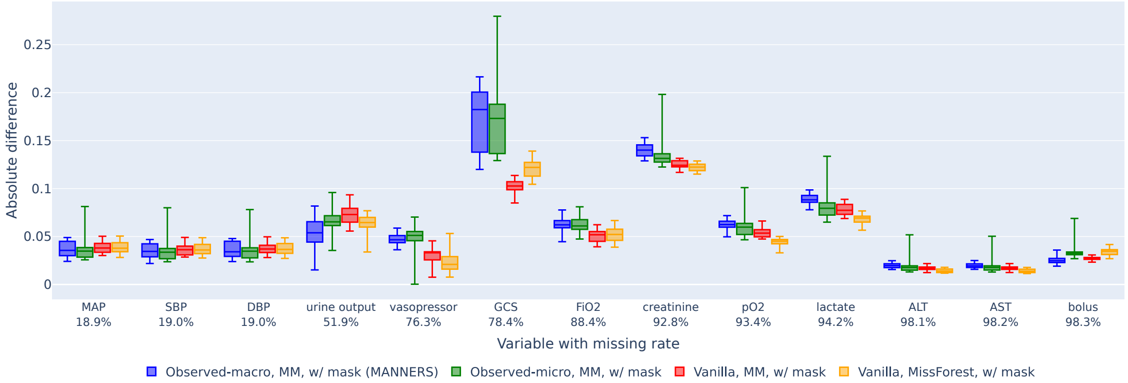

Figure 4. Synthetic variable missing rate deviation

Absolute difference between synthetic and test set missing rate per variable across 20 VAE training runs for four training configurations with mask encoding: observed-macro loss normalization and MM (mean/median) imputation (MANNERS) (blue), observed-micro loss normalization and MM imputation (green), vanilla loss normalization with MM imputation (red), and loss normalization with MissForest imputation (yellow). The box represents the interquartile range (IQR), the line inside the box indicates the median, and the whiskers extend to the most extreme data points. The missing rate across the whole dataset is given per variable, and variables are sorted by ascending missing rate. Lower is better.

showcasing that sole imputation with more complex methodology is not feasible when high missing rates are present. These findings are in line with other studies.[33–36] In missing mask reconstruction, all training configurations with the mask encoding of MANNERS step 1 performed well, with a balanced accuracy of 88% or higher. Additionally, no relationship between missing rate and balanced accuracy could be observed. This is remarkable, as large missing rates directly impose stricter class imbalance, making the mask reconstruction task harder. This could be a case where patterns between missing and observed data are encoded into the learned representations, facilitating the mask reconstruction. Although reconstruction is only a surrogate task in representation learning, it is crucial when combining the trained models with more complex architectures.[46] Additionally, mask encoding enables the reconstruction of incomplete data, maintaining these patterns between missing and observed data.

In the second experiment, we conducted survival and length-of-stay prediction downstream tasks based on frozen latent representations with different downstream model types. AEs trained with full MANNERS showed the overall best results across all downstream tasks and model types. However, the loss masking and rebalancing of MANNERS steps 2 and 3 yielded only marginal gains. Instead, the mask encoding of step 1 contributed the largest improvements. This is in accordance with findings from Paterakis et al.[35] Similar to the first experiment, the imputation method has little effect on MANNERS. This is in stark contrast to the state-of-the-art imputation-only approach, where the most complex and computationally demanding MissForest approach outperformed the other imputation methods. UMAPs with MANNERS showed more expressive clustering of non-survivors and clearer transitions for length of stay, directly informing the improved performance. Crucially, a clinically meaningful and clearly visible relationship between the two external survival and length-of-stay variables and the missing rate of the internal vasopressor variable is present in representations created with MANNERS. This follows results from Tan et al.[8] and highlights that missingness can contain information useful for downstream tasks and is encoded into representations that are learned with MANNERS.

In the last experiment, we evaluated the synthetic data generation capabilities of VAEs trained with MANNERS in comparison to the other configurations. The mask encoding of MANNERS step 1 enables the generation of incomplete synthetic data, and mask quality was similar across configurations. MANNERS significantly outperformed VAEs trained with the state-of-the-art imputation-only approach in data distribution quality for all variables. Again, this highlights the importance of the loss masking of MANNERS step 2. Comparing macro and micro loss averaging, we see that macro averaging again achieves much better results for variables with missing rates above 90%, underlining the importance of step 3 in this scenario. In accordance with the previous two experiments, the imputation method had little effect on VAEs trained with MANNERS. Moreover, we could show that VAEs with MANNERS preserve inter-dataset correlations with the highest fidelity, largely independent of the utilized imputation method. Comparing the results to those using observed-micro loss normalization, we see that the macro averaging of MANNERS step 3 is important for inter-variable correlation preservation. Interestingly, VAEs trained without loss masking even show a decrease in fidelity for the more complex MICE and MissForest imputation techniques compared with the simple MM imputation. We hypothesize the poorer results configuration to be caused by bias introduced by the more advanced imputation methods, creating potentially faulty patterns between observed and imputed data points that leak into the synthetic data.

In summary, we observed that sole imputation techniques do not suffice in the case of very high missing rates and that adjustments to the training pipeline—such as the MANNERS approach—are necessary. Moreover, underlying patterns between missing and observed data contain information and should be encoded into the learned representations. By ablating components of the MANNERS approach, we found that different steps hold varying importance for performance increase,

CellPress
OPEN ACCESS

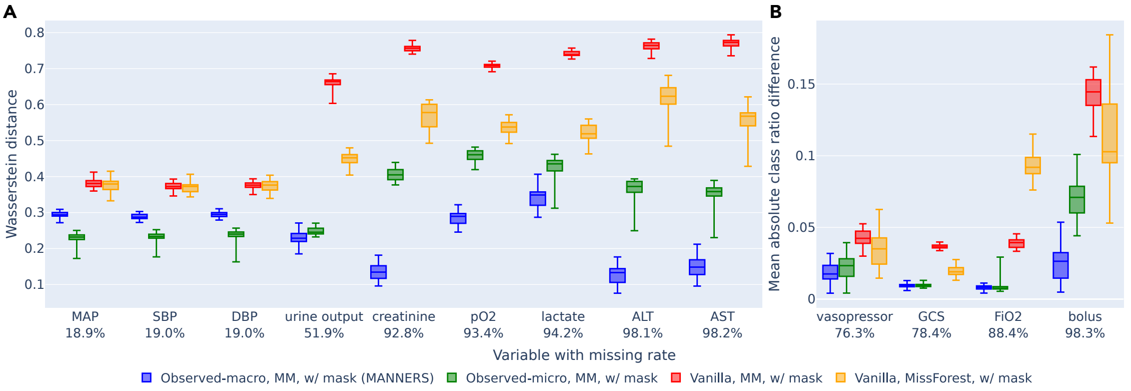

Figure 5. Synthetic data distribution distances

Distance metrics between synthetic data and test set per variable across 20 VAE training runs for four training configurations with mask encoding: observed-macro loss normalization and MM (mean/median) imputation (MANNERS) (blue), observed-micro loss normalization and MM imputation (green), vanilla loss normalization with MM imputation (red), and loss normalization with MissForest imputation (yellow). The box represents the interquartile range (IQR), the line inside the box indicates the median, and the whiskers extend to the most extreme data points. The missing rate across the whole dataset is given per variable, and variables are sorted by ascending missing rate.

(A) Wasserstein distance[56] per metric variable; lower is better.

(B) Mean absolute difference in class fractions for ordinal class variables; lower is better.

dependent on the task with full MANNERS always achieving the best results. Confirming the results of previous studies, missing mask encoding yielded the highest performance increase for downstream classification and regression tasks. Loss masking at missing data points and rebalancing were identified as crucial for reconstructing and synthesizing data with very high missing rates. For models trained with MANNERS, we found no large performance difference between imputation techniques, with MM imputation even performing slightly better in some scenarios. For this reason and the almost 100-fold increase in imputation runtime, we advocate for this rather simple imputation technique in combination with MANNERS. This way, potential bias introduced by more complex imputation methods, as well as computation time, can be avoided. Going forward, MANNERS constitutes a simple, effective, and efficient strategy for handling high missing data rates that can be readily applied to any reconstruction-based representation learning task.

Limitations

Due to the scope of this work, experiments were conducted with only one architecture setup: a fully convolutional AE. Looking forward, it would be interesting to see the impact of MANNERS on more complex architectures, such as transformers. Although we evaluated our approach on three different tasks, we used only one dataset, which could be extended in future publications, e.g., to non-temporal datasets. Additionally, missing data imputation methods accounting for temporality, e.g., spline interpolation, could be analyzed.[58] Moreover, we did not evaluate deep learning imputation methods in this work, and this could be interesting research. However, the results presented in this work show that more complex imputation techniques do not result in significantly better, and sometimes even worse, experiment results of the trained AEs. Additionally, several other studies found that the imputation performance of deep learning models does not exceed that of traditional machine learning approaches.[33–36] Further, we never analyzed the actual imputation performance of the employed methods, and this performance might be impactful for the results discussed in this work. However, we deliberately did so because we are interested in solving the tasks succeeding the imputation and not the imputation itself. Additionally, perturbing parts of the data, as required for the imputation performance analysis, distorts the patterns between missing and observed data and would remove the informative missingness character of the dataset. In the hypotension dataset we chose, variable missing rates jumped from 19% (DBP) to 52% (urine output) to 93% (creatinine), where MANNERS showed slight performance improvements for the first and drastic reconstruction and synthesis performance increases for the latter two. For future work, it would be interesting to use other EHR datasets or other data domains to investigate at which missing rates an application of MANNERS is beneficial. MANNERS extracts information from patterns between observed and missing data and therefore leverages MAR or MNAR missingness mechanisms. It is not clear whether MANNERS would yield performance increases for data with the MCAR mechanism. For this analysis, MANNERS could be evaluated on perturbed complete data using packages such as MissMecha[59] or pyampute[60] with different missingness mechanisms in the future.

METHODS

Mask encoding, value loss masking, and normalization strategy

We introduce MANNERS, a strategy that can be applied to reconstruction tasks for representation learning, e.g., AE training, in multivariate data analysis settings with large amounts of missing data. The starting point is an elementwise boolean indicating observed data points, which is called the missing mask, and an arbitrary elementwise value loss, e.g., squared error. In step 1, reconstruction of the missing mask

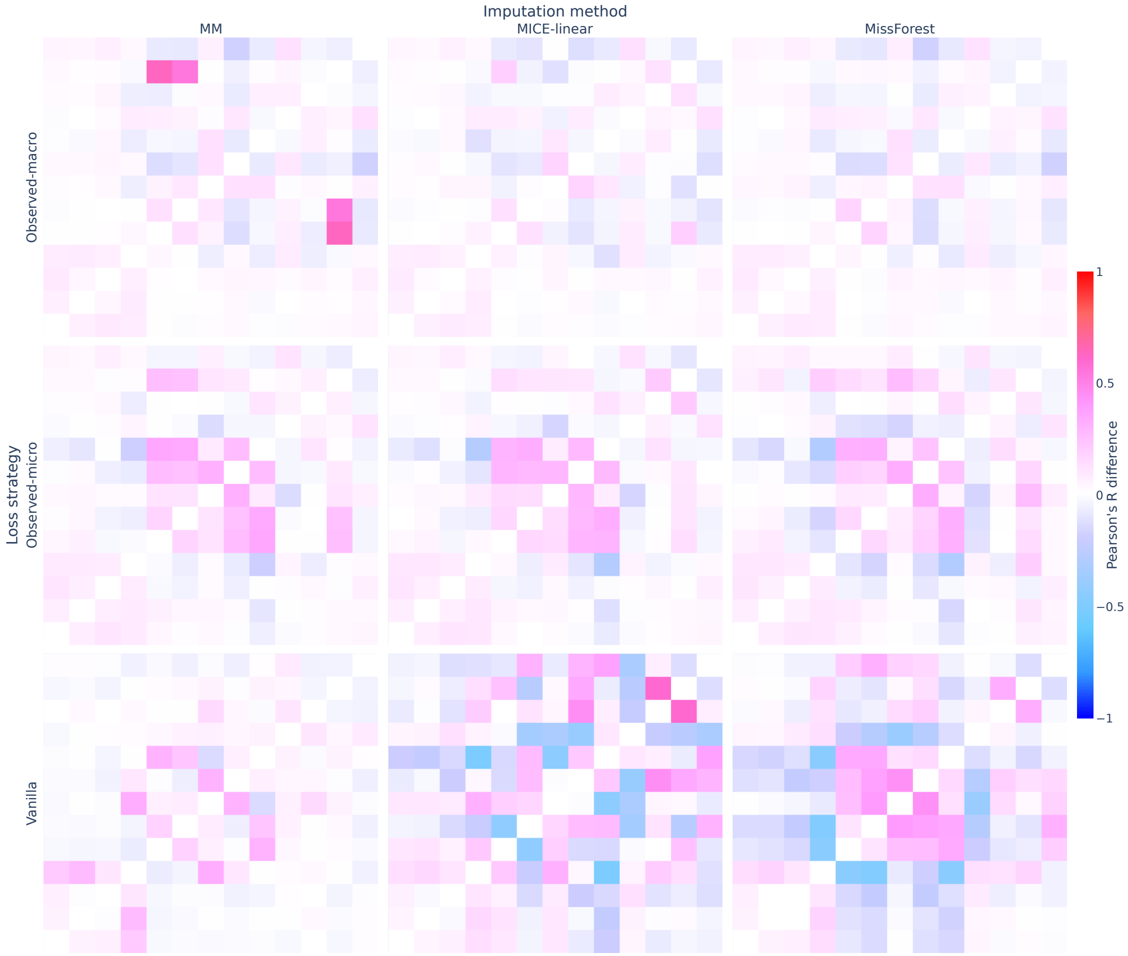

Figure 6. Correlation difference between the synthetic and the original dataset

Signed difference between Pearson correlation coefficients[57] of the full original dataset and stacked synthetic datasets generated by 20 VAEs trained using nine different configurations, with the loss normalization indicated by the row and imputation method indicated by the column. All configurations use mask encoding.

is added to the training objective via binary cross-entropy. In step 2, the value loss is evaluated only on observed data points based on the missing mask. In step 3, this adjusted value loss is normalized using macro averaging. In this way, MANNERS achieves three things: firstly, missing data points with potentially faulty imputations do not influence the training objective. Secondly, the signal of variables with very high missing rates is not lost in the overall training objective due to the macro averaging approach. Lastly, missingness encoding is added to the training objective using the missing mask reconstruction.

Let L be an elementwise value loss tensor of order $k \geq 2$, where the first index denotes the batch dimension and the second index the channel dimension. In our case, the channels represent the 13 time-resolved variables of the hypotension dataset described below, and $k = 3$. Let M be a missing mask of the same order as L, with the same dimensionality per index indicating whether the original entry was observed. Let P be the elementwise reconstruction of M. Then, the binary cross-entropy missing mask reconstruction loss is given as

$$L_{BCE}(M,P) = -\frac{1}{\sum_{i_1}\cdots\sum_{i_k} 1}\sum_{i_1}\cdots\sum_{i_k} M_{i_1,\ldots,i_k}\, log\left(P_{i_1,\ldots,i_k}\right) + \left(1-M_{i_1,\ldots,i_k}\right)\left(1-log\left(P_{i_1,\ldots,i_k}\right)\right).$$

Let $L' = L \odot M$ be the missing adjusted elementwise value loss, which evaluates to 0 for all missing data points. Let $s(X)$ be the sample- and channelwise summation of a tensor X of order k with

$$s(X)_{b,c} = \begin{cases} \sum_{i_3}\cdots\sum_{i_k} X_{b,c,i_3,\ldots,i_k} & \text{if } k > 2 \\ X_{b,c} & \text{else} \end{cases}.$$

Then, let D be the missing adjusted denominator with

$$D_{b,c} = \begin{cases} s(M)_{b,c} & \text{if } s(M)_{b,c} \neq 0 \\ 1 & \text{else} \end{cases}.$$

Then, the sample- and channelwise value loss is given as $L^*_{b,c} = s(L')_{b,c}/D_{b,c}$. Finally, we calculate the mean of L^* over all non-missing sample- and channelwise value loss elements and calculate the weighted sum of value and mask reconstruction

CellPress
OPEN ACCESS

Algorithm 1. Mask encoding, value loss masking, and normalization with MANNERS

function MANNERS(L,M,P,w_{value}, w_{mask}) ▹ L, M, P of order $k \geq 2$
  $L' \leftarrow L \odot M$ ▹mask loss at missing data points
  $k' \leftarrow k$
  $D \leftarrow M$
  while $k' > 2$ do
    $L' \leftarrow \sum_{k'} L'$
    $D \leftarrow \sum_{k'} D$
    $k' \leftarrow k' - 1$
  end while
  $D^* \leftarrow 1_{D \neq 0}$ ▹indicates if some observed data exist for the variable in the batch
  $D \leftarrow max(D,1)$ ▹variable with only missing data gets a denominator of 1
  $L^* \leftarrow L'/D$
  $L_{value} \leftarrow \sum L^* / \sum D^*$
  $L_{mask} \leftarrow L_{BCE}(M,P)$.
  $L_{MANNERS} \leftarrow w_{value} L_{value} + w_{mask} L_{mask}$
  return $L_{MANNERS}$
end function

loss with weights w_{value} and w_{mask}. Hence, the MANNERS loss is given by

$$L_{MANNERS} = w_{value} \frac{\sum_b \sum_c L^*_{b,c}}{\sum_b \sum_c 1_{s(M) \neq 0_{b,c}}} + w_{mask} L_{BCE}(M, P).$$

Note that applying MANNERS loss masking and normalization equals loss averaging when no missing data are present. Further, MANNERS can also be applied to multiple value losses individually. The procedure is given as pseudocode in Algorithm 1.

Dataset and preprocessing

For all experiments, we used a multivariate time-series dataset defined by Gottesman et al.[48] considering the management of acutely hypotensive patients who were treated in the ICU of Beth Israel Deaconess Medical Center, Boston, MA, USA. The original dataset defined by Gottesman et al. was extracted from the MIMIC-III database[61] and used for decision support development[48] and synthetic data generation.[40] Patient ICU stays were added to the dataset if they had at least 7 MAP measurements of 65 mmHg or lower, and data were gathered for the first 48 h of their stay. Using this cohort definition, we extracted 29,439 ICU admissions from the MIMIC-IV v.2.2[49] database. In addition to MAP, DBP, and SBP, serum creatinine, lactate, urine output, pO_2, AST, ALT, and GCS[50] measurements were extracted. Additionally, 3 treatment variables for fraction of inspired oxygen used for ventilation (FiO_2), vasopressor infusion, and administered fluid boluses were added. All treatment variables and the GCS variable were treated as ordinal class variables with 3 classes for vasopressor and fluid boluses, 10 classes for FiO_2, and 13 classes for GCS. All other variables were treated as metric variables. All variables were quantized at every hour, and padding with missing values was applied if patients stayed less than 48 h in the ICU. Metric variables were preprocessed with a Box-Cox transformation[62] and then standardized. Ordinal class variables were standardized for model input. For loss calculation and evaluation, the original, non-standardized class indices were used. For each patient, a binary missing mask was added, indicating observed data points for all 13 variables and all 48 time steps. In addition to these 13 time-resolved variables and their missing mask, we extracted the length of stay in a fraction of days and a binary label for survival during the first 48 h of admission from the MIMIC-IV database. These two non-temporal labels were treated as external data and not used for the initial reconstruction training objective. For comparison, missing data points were imputed using three different approaches: with the first approach, which we call MM imputation, we set all missing data points to the mean for metric variables and to the median class for ordinal class variables. Here, the mean and median were calculated for the training set of the respective split. With the second approach, which we call MICE-linear, we applied MICE paired with Bayesian ridge regression,[43] which was fitted to the training set. In the third approach, we used the MissForest imputation method,[26] which was again fitted on the training set. Afterward, imputation was applied to training, validation, and test sets for each data split and imputation method.

Model architecture and training setup

We used a fully convolutional AE with 3 encoder and 3 decoder layers and applied GELU activation[55] followed by batch normalization between layers. The latent representations were flattened and have a dimension of 256. For setups with the mask encoding of MANNERS step 1, 2D convolutional layers are used, considering both the temporal and value/mask dimension. For the other setups, 1D convolution along the temporal axis is applied. As shown in Figure 7A, variable values and their missing masks were stacked for mask encoding and resulted in a 2D structure along with the temporal dimension. The 13 variables were treated as channels, and convolution was applied to the 2D structure of the temporal and mask dimensions.

For ordinal classification, we used the CORAL framework[63] to ensure rank monotonicity. For regression of discrete variables,

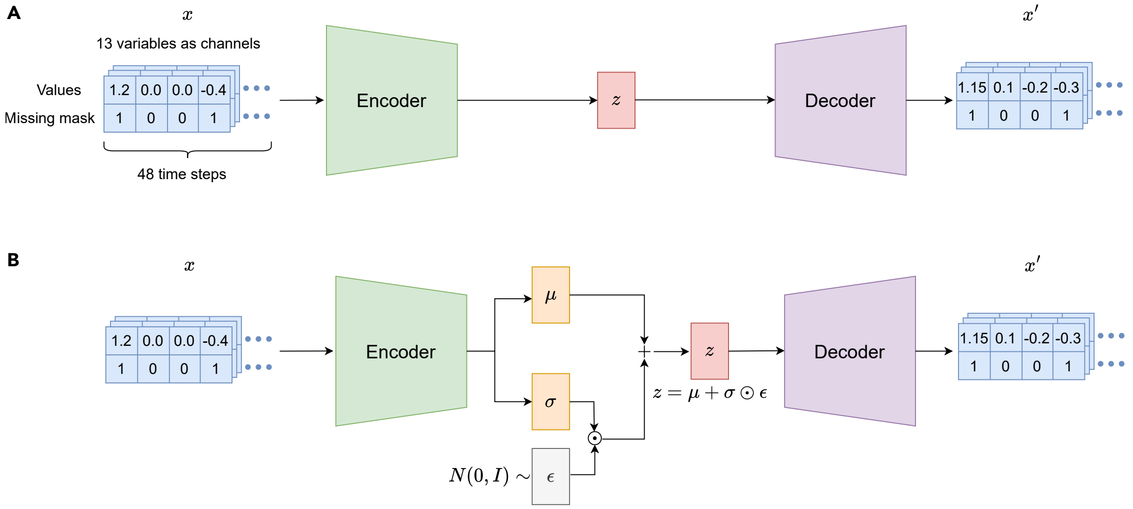

Figure 7. Convolutional autoencoder architecture with mask encoding

Input data point x and reconstruction x'. An input data point has three dimensions: one channel dimension with 13 variables, one temporal dimension with 48 time steps, and one dimension for stacked values and missing mask.

(A) (Un-variational) autoencoder with latent representation z.

(B) Variational autoencoder with latent mean μ and variance σ.[47] Noise ϵ is sampled from a standard Gaussian distribution following the reparameterization trick.

we applied the Huber loss with a δ of 1.0.[64] CORAL, Huber, and missing mask binary cross-entropy loss were all batch weighted and contributed with equal weight to the overall training objective. For configurations without mask encoding, the mask reconstruction loss was omitted. For configurations with loss masking of MANNERS step 2, loss masking and normalization (micro or macro averaging) were applied to the Huber and CORAL elementwise losses individually.

For the synthetic data generation task, we implemented a VAE[47,65] using the same architecture. As depicted in Figure 7B, we duplicated the last encoder layer, generating mean μ and variance σ instead of a single latent representation and finally deriving z by the addition of noise ϵ following the reparameterization trick.[47] Minimizing the Kullback-Leibler divergence[66] to an isotropic Gaussian kernel was added to the training objective.

We used Monte Carlo cross-validation to generate 20 random splits of 70% training, 10% validation, and 20% test set size. On each split, we trained 18 AEs and 18 VAEs using training configurations combining the three imputation methods, implementing or omitting mask encoding, and observed-macro (MANNERS steps 2 and 3), observed-micro (only 2), and vanilla (neither 2 nor 3) loss normalization. We trained all models with a batch size of 64 for a maximum of 200,000 iterations or until an early stopping criterion was reached, which we defined as an increase in a validation loss for 40 epochs. For AEs, this validation loss was the training objective evaluated on the validation set. For VAEs, we generated a synthetic dataset of 30,000 patients. For metric variables, we calculated the percentiles; for ordinal class variables, we calculated class fractions; and from the missing mask, we derived variable missing rates. For each of the three metrics, we calculated the absolute difference between the synthetic dataset and the validation set. Finally, the validation loss for VAEs was the sum of these absolute differences. Additionally, we used a decaying learning rate scheduler,[67] which reduced the initial learning rate of 0.001 by a factor of 0.5 when the validation loss did not decrease by a factor of 0.9999 for 10 epochs.

RESOURCE AVAILABILITY

Lead contact

Requests for further information and resources should be directed to and will be fulfilled by the lead contact, Louis Bellmann (l.bellmann@uke.de).

Materials availability

This study did not generate new materials.

Data and code availability

- The MIMIC-IV v.2.2 database is publicly available and can be requested at https://physionet.org/content/mimiciv/2.2/.
- All original code for MANNERS, hypotension dataset extraction, data preparation, and model training can be accessed in one code repository[68] at https://github.com/UKEIAM/manners or https://doi.org/10.5281/zenodo.19111199.
- Any additional information required to reanalyze the data reported in this paper is available from the lead contact upon request.

ACKNOWLEDGMENTS

This work did not receive specific funding. The authors thank Sara Tiedemann for her support during manuscript preparation and Hümeyra Husseini-Wüsthoff for creating the cover art.

AUTHOR CONTRIBUTIONS

Conceptualization, L.B.; methodology, M.N. and L.B.; investigation, L.B.; writing – original draft, L.B.; writing – review & editing, M.N. and P.B.; supervision, P.B.

DECLARATION OF INTERESTS

The authors declare no competing interests.

CellPress
OPEN ACCESS

DECLARATION OF GENERATIVE AI AND AI-ASSISTED TECHNOLOGIES IN THE WRITING PROCESS

During the preparation of this work, the authors used ChatGPT in order to improve the readability and language of the manuscript. After using this tool, the authors reviewed and edited the content as needed and take full responsibility for the content of the published article.

SUPPLEMENTAL INFORMATION

Supplemental information can be found online at https://doi.org/10.1016/j.patter.2026.101543.

Received: June 25, 2025
Revised: December 16, 2025
Accepted: March 25, 2026
Published: April 23, 2026

REFERENCES

1. Little, T.D., Jorgensen, T.D., Lang, K.M., and Moore, E.W.G. (2014). On the Joys of Missing Data. J. Pediatr. Psychol. *39*, 151–162. https://doi.org/10.1093/jpepsy/jst048.
2. Cismondi, F., Fialho, A.S., Vieira, S.M., Reti, S.R., Sousa, J.M.C., and Finkelstein, S.N. (2013). Missing data in medical databases: Impute, delete or classify? Artif. Intell. Med. *58*, 63–72. https://doi.org/10.1016/j.artmed.2013.01.003.
3. Honaker, J., and King, G. (2010). What to Do about Missing Values in Time-Series Cross-Section Data. Am. J. Pol. Sci. *54*, 561–581. https://doi.org/10.1111/j.1540-5907.2010.00447.x.
4. Penone, C., Davidson, A.D., Shoemaker, K.T., Di Marco, M., Rondinini, C., Brooks, T.M., Young, B.E., Graham, C.H., and Costa, G.C. (2014). Imputation of missing data in life-history trait datasets: which approach performs the best? Methods Ecol. Evol. *5*, 961–970. https://doi.org/10.1111/2041-210X.12232.
5. Bergé, J., Pires, S., Baghi, Q., Touboul, P., and Métris, G. (2015). Dealing with missing data: An inpainting application to the MICROSCOPE space mission. Phys. Rev. D *92*, 112006. https://doi.org/10.1103/PhysRevD.92.112006.
6. Harutyunyan, H., Khachatrian, H., Kale, D.C., Ver Steeg, G., and Galstyan, A. (2019). Multitask learning and benchmarking with clinical time series data. Sci. Data *6*, 96. https://doi.org/10.1038/s41597-019-0103-9.
7. Ullah, S., Xu, Z., Wang, H., Menzel, S., Sendhoff, B., and Bäck, T. (2020). Exploring Clinical Time Series Forecasting with Meta-Features in Variational Recurrent Models. In 2020 International Joint Conference on Neural Networks (IJCNN), pp. 1–9. https://doi.org/10.1109/IJCNN48605.2020.9207254.
8. Tan, A.L.M., Getzen, E.J., Hutch, M.R., Strasser, Z.H., Gutiérrez-Sacristán, A., Le, T.T., Dagliati, A., Morris, M., Hanauer, D.A., Moal, B., et al. (2023). Informative Missingness: What can we learn from patterns in missing laboratory data in the electronic health record? J. Biomed. Inform. *139*, 104306. https://doi.org/10.1016/j.jbi.2023.104306.
9. Denny, J.C. (2012). Mining electronic health records in the genomics era. PLoS Comput. Biol. *8*, e1002823. https://doi.org/10.1371/journal.pcbi.1002823.
10. Zainuddin, A., Hairuddin, M.A., Yassin, A.I.M., Latiff, Z.I.A., and Azhar, A. (2022). Time Series Data and Recent Imputation Techniques for Missing Data: A Review. In 2022 International Conference on Green Energy, Computing and Sustainable Technology (GECOST), pp. 346–350. https://doi.org/10.1109/GECOST55694.2022.10010499.
11. Getzen, E., Ungar, L., Mowery, D., Jiang, X., and Long, Q. (2023). Mining for equitable health: Assessing the impact of missing data in electronic health records. J. Biomed. Inform. *139*, 104269. https://doi.org/10.1016/j.jbi.2022.104269.
12. Morid, M.A., Sheng, O.R.L., and Dunbar, J. (2023). Time Series Prediction Using Deep Learning Methods in Healthcare. ACM Trans. Manage. Inf. Syst. *14*, 1–29. https://doi.org/10.1145/3531326.
13. WEST, M. (1997). Time series decomposition. Biometrika *84*, 489–494. https://doi.org/10.1093/biomet/84.2.489.
14. Knight, M.I., Nason, G.P., and Nunes, M.A. (2017). A wavelet lifting approach to long-memory estimation. Stat. Comput. *27*, 1453–1471. https://doi.org/10.1007/s11222-016-9698-2.
15. Dong, Y., Jiang, H., Wang, X., Mu, M., and Jiang, W. (2024). An interpretable multiscale lifting wavelet contrast network for planetary gearbox fault diagnosis with small samples. Reliab. Eng. Syst. Saf. *251*, 110404. https://doi.org/10.1016/j.ress.2024.110404.
16. Wang, X., Jiang, H., Zeng, T., and Dong, Y. (2026). An adaptive fused domain-cycling variational generative adversarial network for machine fault diagnosis under data scarcity. Inf. Fusion *126*, 103616. https://doi.org/10.1016/j.inffus.2025.103616.
17. Liu, Z., Alavi, A., Li, M., Zhang, X., Liu, Z., Alavi, A., Li, M., and Zhang, X. (2023). Self-Supervised Contrastive Learning for Medical Time Series: A Systematic Review. Sensors *23*, 4221. https://doi.org/10.3390/s23094221.
18. Dong, Y., Jiang, H., Wang, X., and Mu, M. (2025). An interpretable integration fusion time-frequency prototype contrastive learning for machine fault diagnosis with limited labeled samples. Inf. Fusion *124*, 103340. https://doi.org/10.1016/j.inffus.2025.103340.
19. Fan, Y., Feng, C., Wu, R., Liu, C., and Jiang, D. (2024). Multiscale-attention masked autoencoder for missing data imputation of wind turbines. Knowl. Base Syst. *299*, 112114. https://doi.org/10.1016/j.knosys.2024.112114.
20. Mendonça Freire, G., and Curi, M. (2024). Masked Autoencoder Transformer for Missing Data Imputation of PISA. In Artificial Intelligence in Education. Posters and Late Breaking Results, Workshops and Tutorials, Industry and Innovation Tracks, Practitioners, Doctoral Consortium and Blue Sky, A.M. Olney, I.A. Chounta, Z. Liu, O.C. Santos, and I.I. Bittencourt, eds. (Springer Nature Switzerland), pp. 364–372. https://doi.org/10.1007/978-3-031-64315-6_33.
21. Liu, Q., Ye, J., Liang, H., Sun, L., and Du, B. (2025). TS-MAE: A masked autoencoder for time series representation learning. Inf. Sci. *690*, 121576. https://doi.org/10.1016/j.ins.2024.121576.
22. Wu, H., Xu, J., Wang, J., and Long, M. (2021). Autoformer: Decomposition Transformers with Auto-Correlation for Long-Term Series Forecasting. In Advances in Neural Information Processing Systems, *34* (Curran Associates, Inc.), pp. 22419–22430.
23. Wang, X., Liu, H., Du, J., Yang, Z., and Dong, X. (2023). CLformer: Locally grouped auto-correlation and convolutional transformer for long-term multivariate time series forecasting. Eng. Appl. Artif. Intell. *121*, 106042. https://doi.org/10.1016/j.engappai.2023.106042.
24. Azur, M.J., Stuart, E.A., Frangakis, C., and Leaf, P.J. (2011). Multiple imputation by chained equations: what is it and how does it work? Int. J. Methods Psychiatr. Res. *20*, 40–49. https://doi.org/10.1002/mpr.329.
25. Khan, S.I., and Hoque, A.S.M.L. (2020). SICE: an improved missing data imputation technique. J. Big Data *7*, 37. https://doi.org/10.1186/s40537-020-00313-w.
26. Stekhoven, D.J., and Bühlmann, P. (2012). MissForest—non-parametric missing value imputation for mixed-type data. Bioinformatics *28*, 112–118. https://doi.org/10.1093/bioinformatics/btr597.
27. Yoon, J., Jordon, J., and Schaar, M. (2018). GAIN: Missing Data Imputation using Generative Adversarial Nets. In Proceedings of the 35th International Conference on Machine Learning (PMLR), pp. 5689–5698.
28. Bernardini, M., Doinychko, A., Romeo, L., Frontoni, E., and Amini, M.R. (2023). A novel missing data imputation approach based on clinical conditional Generative Adversarial Networks applied to EHR datasets. Comput. Biol. Med. *163*, 107188. https://doi.org/10.1016/j.compbiomed.2023.107188.

29. Gondara, L., and Wang, K. (2018). MIDA: Multiple Imputation Using Denoising Autoencoders. In Advances in Knowledge Discovery and Data Mining, D. Phung, V.S. Tseng, G.I. Webb, B. Ho, M. Ganji, and L. Rashidi, eds. (Springer International Publishing), pp. 260–272. https://doi.org/10.1007/978-3-319-93040-4_21.

30. Ou, C., Zhu, H., Shardt, Y.A.W., Ye, L., Yuan, X., Wang, Y., Yang, C., and Gui, W. (2024). Missing-Data Imputation With Position-Encoding Denoising Autoencoders for Industrial Processes. IEEE Trans. Instrum. Meas. *73*, 1–11. https://doi.org/10.1109/TIM.2024.3443350.

31. Duy Le, T., Beuran, R., and Tan, Y. (2018). Comparison of the Most Influential Missing Data Imputation Algorithms for Healthcare. In 2018 10th International Conference on Knowledge and Systems Engineering (KSE), pp. 247–251. https://doi.org/10.1109/KSE.2018.8573344.

32. KALKAN, Ö.K., Kara, Y., and KELECİOĞLU, H. (2018). Evaluating Performance of Missing Data Imputation Methods in IRT Analyses. International Journal of Assessment Tools in Education *5*, 403–416. https://doi.org/10.21449/ijate.430720.

33. Sun, Y., Li, J., Xu, Y., Zhang, T., and Wang, X. (2023). Deep learning versus conventional methods for missing data imputation: A review and comparative study. Expert Syst. Appl. *227*, 120201. https://doi.org/10.1016/j.eswa.2023.120201.

34. Zhang, C., Chu, X., Ma, L., Zhu, Y., Wang, Y., Wang, J., and Zhao, J. (2022). M3Care: Learning with Missing Modalities in Multimodal Healthcare Data. In Proceedings of the 28th ACM SIGKDD Conference on Knowledge Discovery and Data Mining. KDD '22 (Association for Computing Machinery), pp. 2418–2428. https://doi.org/10.1145/3534678.3539388.

35. Paterakis, G., Fafalios, S., Charonyktakis, P., Christophides, V., and Tsamardinos, I. (2024). Do We Really Need Imputation in AutoML Predictive Modeling? ACM Trans. Knowl. Discov. Data *18*, 1–64. https://doi.org/10.1145/3643643.

36. Costa, A.F., Santos, M.S., Soares, J.P., and Abreu, P.H. (2018). Missing Data Imputation via Denoising Autoencoders: The Untold Story. In Advances in Intelligent Data Analysis XVII, W. Duivesteijn, A. Siebes, and A. Ukkonen, eds. (Springer International Publishing), pp. 87–98. https://doi.org/10.1007/978-3-030-01768-2_8.

37. Rubin, D.B. (1976). Inference and missing data. Biometrika *63*, 581–592. https://doi.org/10.1093/biomet/63.3.581.

38. Yoon, J., Zame, W.R., and van der Schaar, M. (2019). Estimating Missing Data in Temporal Data Streams Using Multi-Directional Recurrent Neural Networks. IEEE Trans. Biomed. Eng. *66*, 1477–1490. https://doi.org/10.1109/TBME.2018.2874712.

39. Tahir, M., Abdullah, A., Udzir, N.I., and Kasmiran, K.A. (2025). A novel approach for handling missing data to enhance network intrusion detection system. Cyber Security and Applications *3*, 100063. https://doi.org/10.1016/j.csa.2024.100063.

40. Kuo, N.I.H., Polizzotto, M.N., Finfer, S., Garcia, F., Sönnerborg, A., Zazzi, M., Böhm, M., Kaiser, R., Jorm, L., and Barbieri, S. (2022). The Health Gym: synthetic health-related datasets for the development of reinforcement learning algorithms. Sci. Data *9*, 693. https://doi.org/10.1038/s41597-022-01784-7.

41. Kuang, S., Huang, Y., and Song, J. (2025). Unsupervised data imputation with multiple importance sampling variational autoencoders. Sci. Rep. *15*, 3409. https://doi.org/10.1038/s41598-025-87641-0.

42. Bank, D., Koenigstein, N., and Giryes, R. (2021). Autoencoders. Preprint at arXiv. https://doi.org/10.48550/arXiv.2003.05991.

43. Michimae, H., and Emura, T. (2022). Bayesian ridge estimators based on copula-based joint prior distributions for regression coefficients. Comput. Stat. *37*, 2741–2769. https://doi.org/10.1007/s00180-022-01213-8.

44. Shadbahr, T., Roberts, M., Stanczuk, J., Gilbey, J., Teare, P., Dittmer, S., Thorpe, M., Torné, R.V., Sala, E., Lió, P., et al. (2023). The impact of imputation quality on machine learning classifiers for datasets with missing values. Commun. Med. *3*, 139. https://doi.org/10.1038/s43856-023-00356-z.

45. Wang, Z., Akande, O., Poulos, J., and Li, F. (2022). Are deep learning models superior for missing data imputation in large surveys? Evidence from an empirical comparison. Survey Methodology, Statistics Canada *48*. http://www.statcan.gc.ca/pub/12-001-x/2022002/article/00009-eng.htm.

46. Rombach, R., Blattmann, A., Lorenz, D., Esser, P., and Ommer, B. (2022). High-Resolution Image Synthesis with Latent Diffusion Models. Preprint at arXiv. https://doi.org/10.48550/arXiv.2112.10752.

47. Kingma, D.P., and Welling, M. (2014). Auto-encoding variational bayes. In 2nd International Conference on Learning Representations (ICLR 2014). https://doi.org/10.48550/arXiv.1312.6114.

48. Gottesman, O., Futoma, J., Liu, Y., Parbhoo, S., Celi, L.A., Brunskill, E., and Doshi-Velez, F. (2020). Interpretable off-policy evaluation in reinforcement learning by highlighting influential transitions. In Proceedings of the 37th International Conference on Machine Learning, H. Daumé and A. Singh, eds. (JMLR.org), pp. 3658–3667. https://doi.org/10.48550/arXiv.2002.03478.

49. Johnson, A.E.W., Bulgarelli, L., Shen, L., Gayles, A., Shammout, A., Horng, S., Pollard, T.J., Hao, S., Moody, B., Gow, B., et al. (2023). MIMIC-IV, a freely accessible electronic health record dataset. Sci. Data *10*, 1. https://doi.org/10.1038/s41597-022-01899-x.

50. Teasdale, G., and Jennett, B. (1974). Assessment of coma and impaired consciousness. A practical scale. Lancet *2*, 81–84. https://doi.org/10.1016/s0140-6736(74)91639-0.

51. Bengio, Y., Yao, L., Alain, G., and Vincent, P. (2013). Generalized Denoising Auto-Encoders as Generative Models. In Proceedings of the 27th International Conference on Neural Information Processing Systems, C.J.C. Burges, L. Bottou, M. Welling, Z. Ghahramani, and K.Q. Weinberger, eds. (Curran Associates, Inc.). https://doi.org/10.48550/arXiv.1305.6663.

52. Zhou, C., and Paffenroth, R.C. (2017). Anomaly detection with robust deep autoencoders. In Proceedings of the 23rd ACM SIGKDD International Conference on Knowledge Discovery and Data Mining (Association for Computing Machinery), pp. 665–674. https://doi.org/10.1145/3097983.3098052.

53. Kendall, M.G. (1938). A New Measure of Rank Correlation. Biometrika *30*, 81–93. https://doi.org/10.2307/2332226.

54. McInnes, L., Healy, J., Saul, N., and Großberger, L. (2018). UMAP: Uniform Manifold Approximation and Projection. J. Open Source Softw. *3*, 861. https://doi.org/10.21105/joss.00861.

55. Hendrycks, D., and Gimpel, K. (2023). Gaussian Error Linear Units (GELUs). Preprint at arXiv. https://doi.org/10.48550/arXiv.1606.08415.

56. Vaseršteĭn, L. (1969). Markov processes over denumerable products of spaces, describing large systems of automata. Probl. Peredachi Inf. *5*.

57. Pearson, K., and Galton, F. (1895). Vii. note on regression and inheritance in the case of two parents. Proc. R. Soc. Lond. *58*, 240–242. https://doi.org/10.1098/rspl.1895.0041.

58. Nickerson, P., Baharloo, R., Davoudi, A., Bihorac, A., and Rashidi, P. (2018). Comparison of Gaussian Processes Methods to Linear methods for Imputation of Sparse Physiological Time Series. Annu. Int. Conf. IEEE Eng. Med. Biol. Soc. *2018*, 4106–4109. https://doi.org/10.1109/EMBC.2018.8513303.

59. Zhou, Y., Bouadjenek, M.R., and Aryal, S. (2025). MissMecha: An All-in-One Python Package for Studying Missing Data Mechanisms. Preprint at arXiv. https://doi.org/10.48550/arXiv.2508.04740.

60. Schouten, R.M., Lugtig, P., and Vink, G. (2018). Generating missing values for simulation purposes: a multivariate amputation procedure. J. Stat. Comput. Simulat. *88*, 2909–2930. https://doi.org/10.1080/00949655.2018.1491577.

61. Johnson, A.E.W., Pollard, T.J., Shen, L., Lehman, L.w.H., Feng, M., Ghassemi, M., Moody, B., Szolovits, P., Celi, L.A., and Mark, R.G. (2016). MIMIC-III, a freely accessible critical care database. Sci. Data *3*, 160035. https://doi.org/10.1038/sdata.2016.35.

CellPress
OPEN ACCESS

62. Box, G.E.P., and Cox, D.R. (1964). An Analysis of Transformations. J. Roy. Stat. Soc. B *26*, 211–243. https://doi.org/10.1111/j.2517-6161.1964.tb00553.x.

63. Cao, W., Mirjalili, V., and Raschka, S. (2020). Rank consistent ordinal regression for neural networks with application to age estimation. Pattern Recognit. Lett. *140*, 325–331. https://doi.org/10.1016/j.patrec.2020.11.008.

64. Huber, P.J. (2004). Robust Statistics (John Wiley & Sons). https://doi.org/10.1002/9780470434697.

65. Kingma, D.P., and Welling, M. (2022). Auto-Encoding Variational Bayes. Preprint at arXiv. https://doi.org/10.48550/arXiv.1312.6114.

66. Kullback, S., and Leibler, R.A. (1951). On Information and Sufficiency. Ann. Math. Statist. *22*, 79–86. https://doi.org/10.1214/aoms/1177729694.

67. Bengio, Y. (2012). Practical Recommendations for Gradient-Based Training of Deep Architectures. In Neural Networks: Tricks of the Trade, Second Edition, G. Montavon, G.B. Orr, and K.R. Müller, eds. (Springer), pp. 437–478. https://doi.org/10.1007/978-3-642-35289-8_26.

68. Bellmann, L. (2026). MANNERS: a strategy for representation learning in multivariate datasets with high proportions of missing data. Zenodo. URL: https://doi.org/10.5281/zenodo.19111199.

Patterns, Volume *7*

Supplemental information

MANNERS: A strategy for representation learning in multivariate datasets with high proportions of missing data

Louis Bellmann, Maximilian Nielsen, and Philipp Breitfeld

1 Supplemental Figures

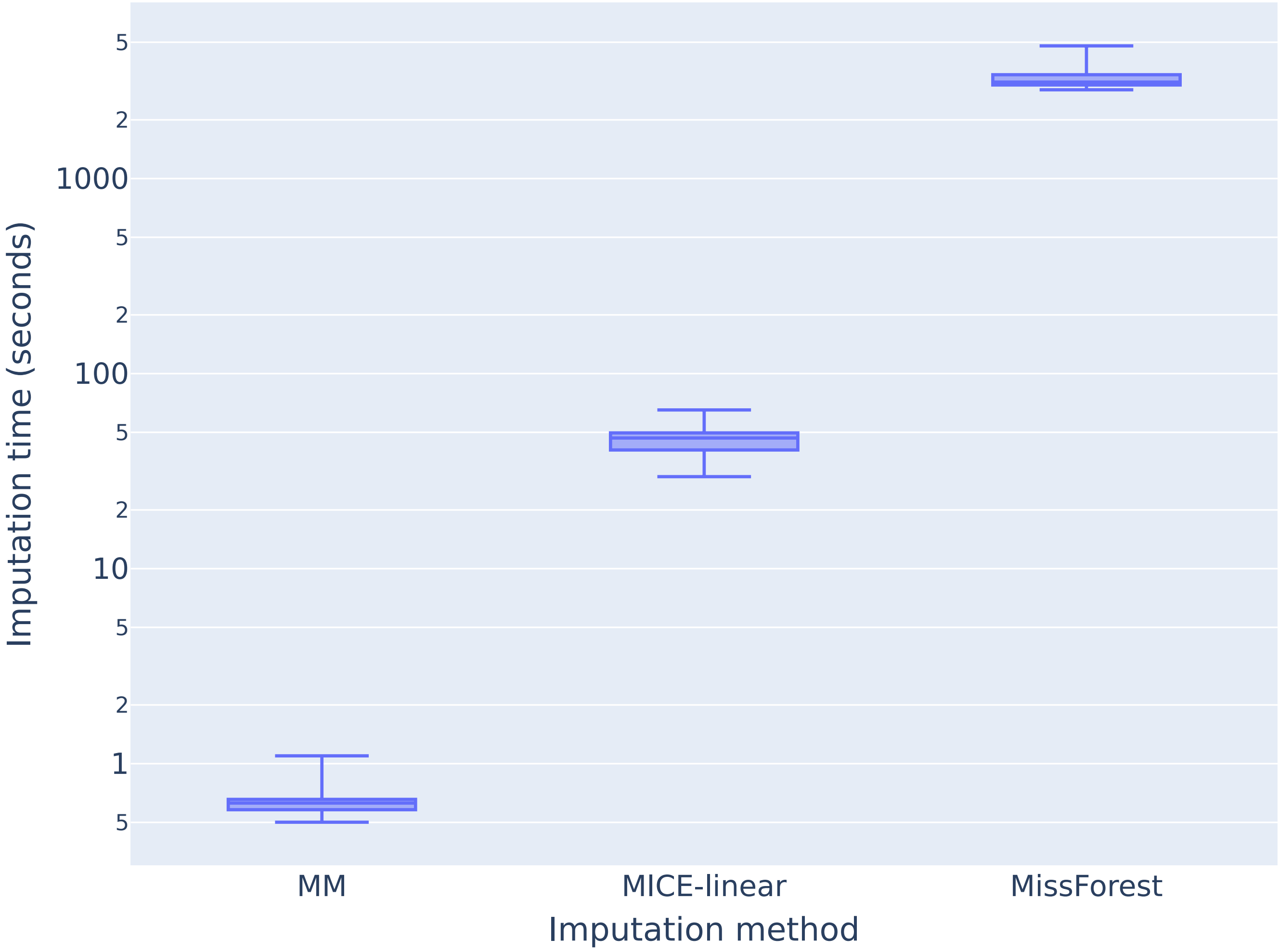

Figure S1: Runtimes per imputation method

Single-threaded runtimes per imputation method in seconds across 720 training runs in logarithmic units. Imputation methods are fitted to the training set and applied to training, validation and test set. The box represents the interquartile range (IQR), the line inside the box indicates the median, and the whiskers extend to the most extreme data points.

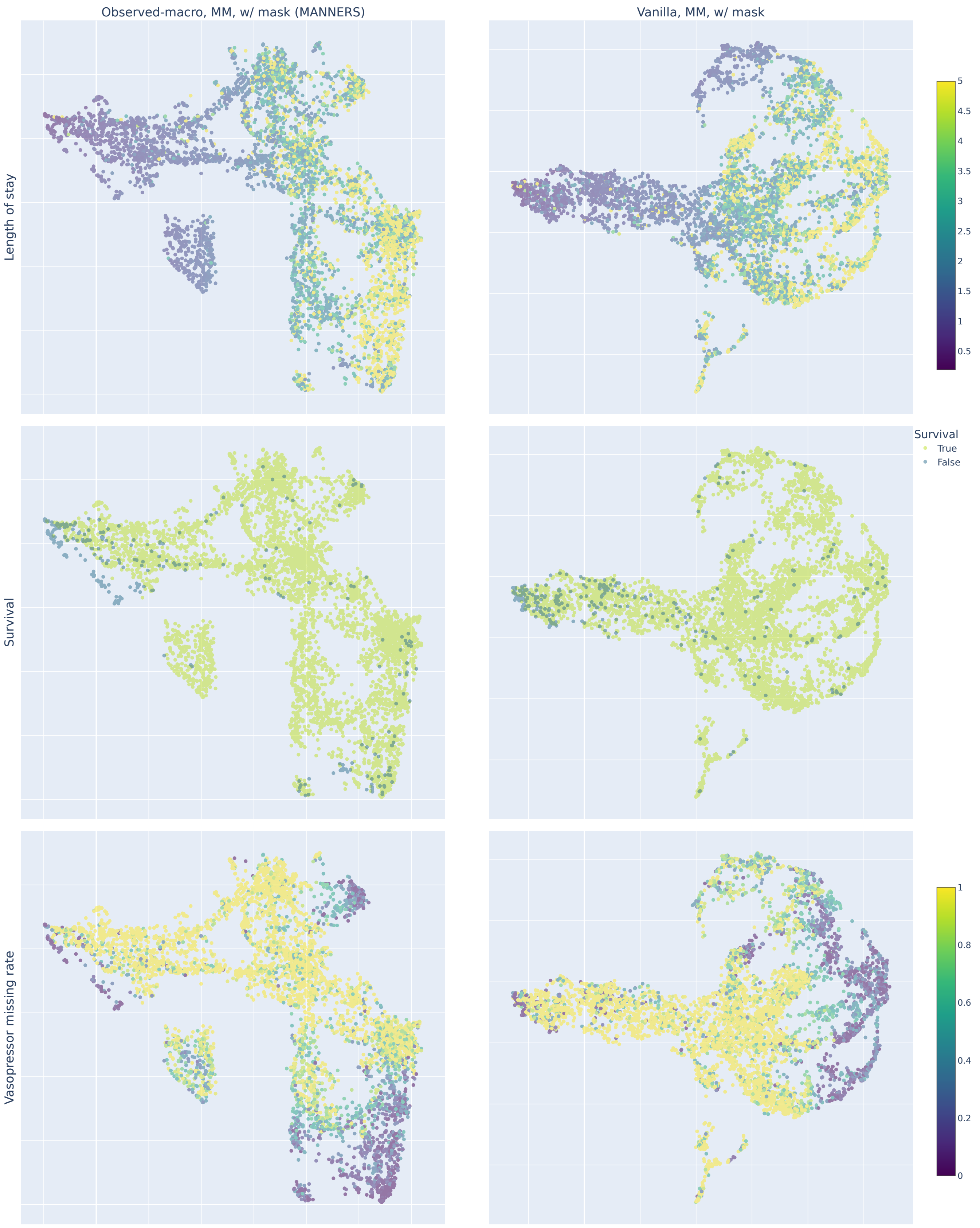

Figure S2: UMAP projections of latent representations

Test set representation projections into $\mathbb{R}^2$ on the first Monte Carlo cross-validation split for the MANNERS configuration with observed-macro loss normalization, MM imputation and mask encoding (left), and vanilla loss normalization, MM imputation and mask encoding (right). Projections are colored by length of ICU stay in fraction of days (top), survival during the first 48 hours (middle), and vasopressor missing rate (bottom).

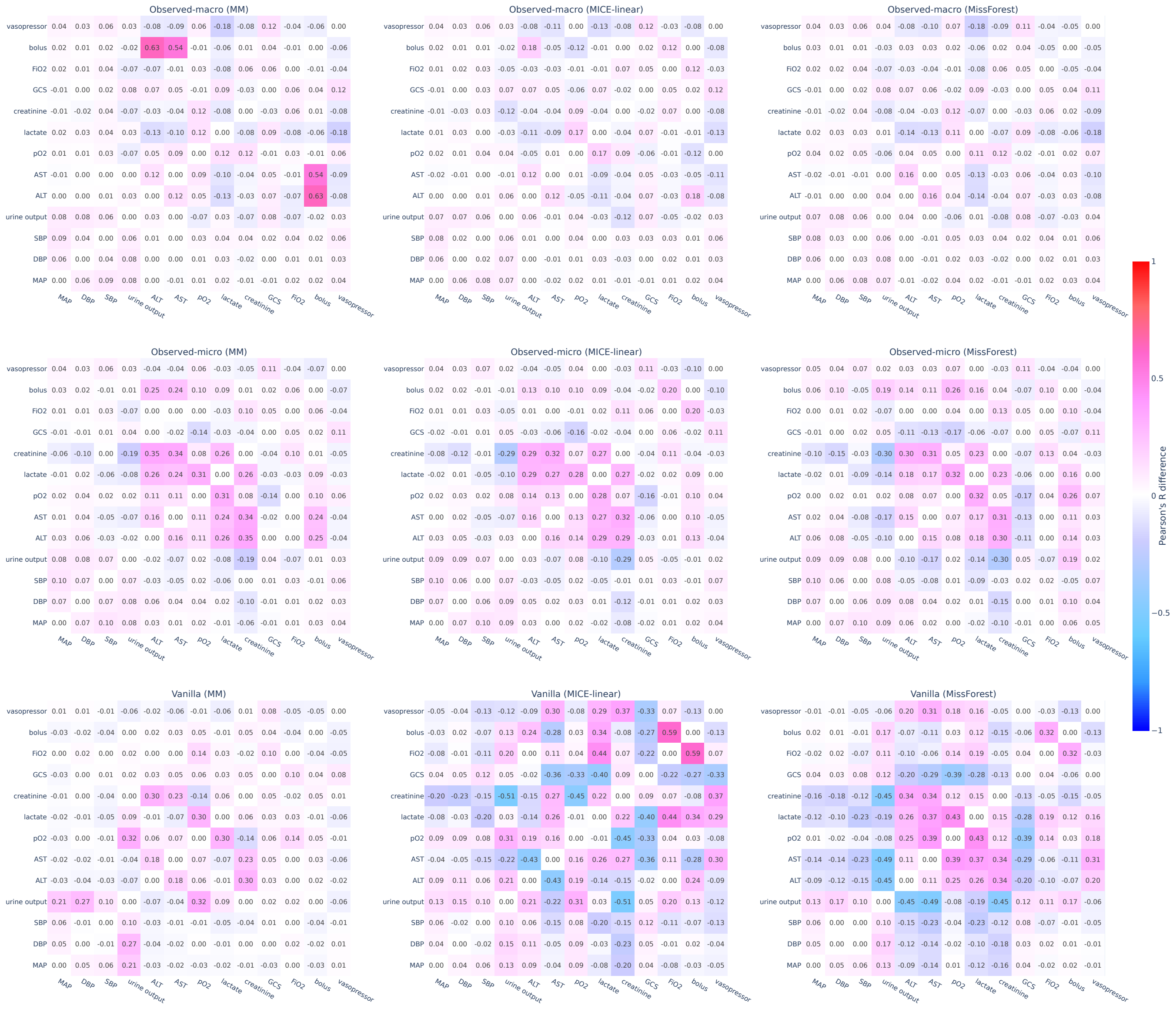

Figure S3: Correlation difference between synthetic and original dataset

Signed difference between Pearson correlation coefficients of the full original dataset and stacked synthetic datasets generated by 20 VAEs trained with observed macro (top), observed micro (middle), and vanilla (bottom) loss normalization strategies. Missing values were imputed with MM (left), linear MICE (middle), and MissForest (right) imputation techniques. All VAEs used missing mask encoding.

2 Supplemental Tables

Table S1: Missing mask reconstruction results

Loss normalization	Variable	MM	MICE-linear	MissForest
observed-macro	MAP	.996 (.0)	.995 (.0)	.995 (.0)
	DBP	.997 (.0)	.996 (.0)	.996 (.001)
	SBP	.997 (.0)	.996 (.0)	.996 (.001)
	urine output	.961 (.003)	.96 (.003)	.962 (.004)
	ALT	.995 (.001)	.995 (.001)	.994 (.001)
	AST	.995 (.001)	.996 (.001)	.995 (.001)
	pO2	.977 (.003)	.976 (.002)	.973 (.003)
	lactate	.977 (.003)	.977 (.002)	.975 (.003)
	creatinine	.991 (.002)	.99 (.003)	.99 (.002)
	GCS	.966 (.003)	.964 (.002)	.966 (.003)
	FiO2	.945 (.006)	.945 (.004)	.943 (.004)
	bolus	.964 (.005)	.965 (.003)	.961 (.004)
	vasopressor	.985 (.002)	.987 (.002)	.988 (.002)
observed-micro	MAP	.997 (.001)	.996 (.0)	.996 (.0)
	DBP	.998 (.0)	.998 (.0)	.998 (.001)
	SBP	.998 (.0)	.998 (.0)	.998 (.0)
	urine output	.961 (.003)	.957 (.004)	.96 (.005)
	ALT	.995 (.001)	.995 (.001)	.995 (.002)
	AST	.996 (.001)	.995 (.001)	.995 (.002)
	pO2	.976 (.002)	.975 (.004)	.973 (.004)
	lactate	.982 (.002)	.981 (.003)	.978 (.004)
	creatinine	.993 (.002)	.993 (.002)	.992 (.003)
	GCS	.965 (.003)	.964 (.004)	.965 (.003)
	FiO2	.944 (.005)	.942 (.006)	.939 (.006)
	bolus	.97 (.004)	.967 (.005)	.969 (.004)
	vasopressor	.986 (.001)	.986 (.001)	.987 (.001)
vanilla	MAP	.996 (.0)	.994 (.0)	.99 (.001)
	DBP	.997 (.0)	.995 (.0)	.991 (.002)
	SBP	.997 (.0)	.995 (.0)	.991 (.002)
	urine output	.981 (.002)	.961 (.003)	.888 (.009)
	ALT	.997 (.001)	.997 (.001)	.991 (.003)
	AST	.998 (.001)	.997 (.001)	.992 (.003)
	pO2	.985 (.001)	.977 (.002)	.958 (.006)
	lactate	.988 (.002)	.977 (.002)	.959 (.006)
	creatinine	.996 (.001)	.992 (.003)	.976 (.005)
	GCS	.995 (.002)	.965 (.002)	.915 (.008)
	FiO2	.979 (.003)	.958 (.004)	.903 (.011)
	bolus	.976 (.005)	.966 (.004)	.956 (.008)
	vasopressor	.992 (.001)	.984 (.001)	.971 (.004)

Balanced accuracy (higher is better) of mask reconstruction on test set per variable. Average and standard deviation across 20 training runs per loss normalization and imputation method. Mask encoding is used for all configurations.

Table S2: Value reconstruction results with encoded mask

Loss normalization	Metric	Variable	MM	MICE-linear	MissForest
observed-macro	MAE	MAP	.195 (.007)	.206 (.008)	.215 (.009)
		DBP	.216 (.008)	.228 (.008)	.235 (.01)
		SBP	.245 (.01)	.252 (.01)	.262 (.014)
		urine output	.295 (.016)	.296 (.012)	.306 (.015)
		ALT	.162 (.011)	.185 (.019)	.207 (.014)
		AST	.159 (.014)	.19 (.017)	.205 (.014)
		pO2	.259 (.004)	.284 (.013)	.297 (.018)
		lactate	.227 (.003)	.248 (.007)	.253 (.019)
		creatinine	.088 (.005)	.162 (.011)	.152 (.023)
	Kendall's tau	GCS	.976 (.002)	.968 (.001)	.966 (.005)
		FiO2	.949 (.007)	.938 (.007)	.925 (.007)
		bolus	.946 (.007)	.937 (.008)	.93 (.008)
		vasopressor	.987 (.001)	.985 (.001)	.985 (.001)
observed-micro	MAE	MAP	.124 (.003)	.124 (.003)	.125 (.003)
		DBP	.152 (.003)	.153 (.004)	.154 (.004)
		SBP	.182 (.007)	.184 (.006)	.188 (.005)
		urine output	.24 (.023)	.251 (.02)	.267 (.02)
		ALT	.404 (.052)	.429 (.056)	.564 (.105)
		AST	.396 (.047)	.416 (.046)	.533 (.096)
		pO2	.466 (.009)	.492 (.009)	.552 (.004)
		lactate	.387 (.018)	.421 (.013)	.486 (.029)
		creatinine	.265 (.012)	.313 (.017)	.316 (.021)
	Kendall's tau	GCS	.976 (.002)	.968 (.004)	.971 (.003)
		FiO2	.943 (.007)	.931 (.007)	.921 (.008)
		bolus	.92 (.01)	.89 (.02)	.881 (.019)
		vasopressor	.993 (.001)	.992 (.001)	.993 (.001)
vanilla	MAE	MAP	.218 (.007)	.282 (.009)	.378 (.027)
		DBP	.243 (.007)	.31 (.008)	.396 (.022)
		SBP	.278 (.012)	.337 (.007)	.414 (.017)
		urine output	.391 (.01)	.398 (.008)	.494 (.021)
		ALT	.801 (.012)	.789 (.018)	.564 (.056)
		AST	.803 (.012)	.75 (.015)	.494 (.072)
		pO2	.644 (.006)	.547 (.009)	.506 (.015)
		lactate	.712 (.014)	.459 (.026)	.502 (.027)
		creatinine	.717 (.039)	.617 (.021)	.57 (.032)
	Kendall's tau	GCS	.96 (.008)	.905 (.003)	.86 (.015)
		FiO2	.873 (.014)	.881 (.01)	.749 (.049)
		bolus	.91 (.012)	.938 (.013)	.976 (.012)
		vasopressor	.976 (.001)	.973 (.003)	.983 (.006)

Test set value reconstruction results for observed data points per variable. Average and standard deviation across 20 training runs per loss normalization and imputation method. Mask encoding is used for all configurations. Performance is measured in mean absolute error (lower is better) for metric variables, and in Kendall's tau rank correlation coefficient (higher is better) for ordinal class variables.

Table S3: Value reconstruction results without encoded mask

Loss normalization	Metric	Variable	MM	MICE-linear	MissForest
observed-macro	MAE	MAP	.127 (.004)	.155 (.005)	.226 (.005)
		DBP	.154 (.005)	.188 (.005)	.264 (.004)
		SBP	.184 (.007)	.234 (.007)	.315 (.004)
		urine output	.201 (.012)	.258 (.007)	.411 (.007)
		ALT	.162 (.007)	.184 (.008)	.545 (.035)
		AST	.16 (.01)	.179 (.009)	.482 (.026)
		pO2	.257 (.016)	.282 (.005)	.402 (.016)
		lactate	.216 (.012)	.25 (.005)	.398 (.02)
		creatinine	.097 (.004)	.181 (.01)	.365 (.016)
	Kendall's tau	GCS	.978 (.001)	.975 (.004)	.93 (.007)
		FiO2	.951 (.007)	.926 (.003)	.802 (.02)
		bolus	.952 (.008)	.921 (.008)	.804 (.015)
		vasopressor	.988 (.001)	.989 (.001)	.973 (.003)
observed-micro	MAE	MAP	.097 (.002)	.099 (.002)	.111 (.006)
		DBP	.106 (.003)	.116 (.003)	.142 (.007)
		SBP	.113 (.005)	.137 (.004)	.18 (.012)
		urine output	.152 (.006)	.196 (.009)	.274 (.036)
		ALT	.392 (.021)	.391 (.02)	.776 (.014)
		AST	.394 (.019)	.382 (.019)	.75 (.018)
		pO2	.404 (.007)	.401 (.006)	.5 (.007)
		lactate	.347 (.007)	.351 (.009)	.542 (.013)
		creatinine	.244 (.004)	.302 (.008)	.596 (.012)
	Kendall's tau	GCS	.978 (.001)	.98 (.007)	.988 (.002)
		FiO2	.939 (.005)	.936 (.004)	.883 (.006)
		bolus	.896 (.006)	.863 (.014)	.371 (.027)
		vasopressor	.996 (.001)	.997 (.0)	.993 (.001)
vanilla	MAE	MAP	.105 (.002)	.139 (.005)	.24 (.025)
		DBP	.113 (.004)	.165 (.005)	.286 (.018)
		SBP	.131 (.005)	.201 (.009)	.334 (.012)
		urine output	.164 (.006)	.209 (.008)	.419 (.037)
		ALT	.813 (.012)	.802 (.016)	.481 (.07)
		AST	.822 (.011)	.766 (.015)	.383 (.076)
		pO2	.664 (.011)	.509 (.011)	.481 (.016)
		lactate	.784 (.007)	.444 (.026)	.457 (.029)
		creatinine	.791 (.013)	.573 (.024)	.498 (.036)
	Kendall's tau	GCS	.98 (.001)	.941 (.004)	.922 (.02)
		FiO2	.962 (.004)	.981 (.003)	.916 (.027)
		bolus	.984 (.003)	.995 (.002)	.995 (.004)
		vasopressor	.994 (.001)	.994 (.001)	.996 (.004)

Test set value reconstruction results for observed data points per variable. Average and standard deviation across 20 training runs per loss normalization and imputation method. Mask encoding is never used. Performance is measured in mean absolute error (lower is better) for metric variables, and in Kendall's tau rank correlation coefficient (higher is better) for ordinal class variables.

Table S4: Downstream survival classification task results

Mask encoded	Loss normalization	Imputation mode	linear	SVM	single layer	MLP
yes	observed-macro	MM	**.858 (.021)**	.841 (.025)	.833 (.022)	.854 (.021)
		MICE-linear	.847 (.021)	**.848 (.024)**	.825 (.023)	.848 (.024)
		MissForest	.852 (.017)	.848 (.026)	**.839 (.017)**	**.858 (.019)**
	observed-micro	MM	.846 (.018)	.829 (.025)	.815 (.021)	.834 (.018)
		MICE-linear	.848 (.018)	.838 (.025)	.819 (.025)	.84 (.022)
		MissForest	.844 (.019)	.838 (.028)	.827 (.021)	.842 (.025)
	vanilla	MM	.837 (.019)	.812 (.026)	.817 (.019)	.839 (.019)
		MICE-linear	.84 (.019)	.833 (.025)	.818 (.023)	.84 (.026)
		MissForest	.825 (.021)	.813 (.025)	.801 (.023)	.822 (.021)
no	observed-macro	MM	.652 (.013)	.652 (.02)	.622 (.016)	.646 (.017)
		MICE-linear	.669 (.018)	.696 (.017)	.626 (.018)	.656 (.018)
		MissForest	.761 (.02)	.744 (.026)	.736 (.024)	.755 (.019)
	observed-micro	MM	.643 (.021)	.659 (.022)	.6 (.014)	.62 (.015)
		MICE-linear	.676 (.021)	.699 (.024)	.632 (.019)	.656 (.022)
		MissForest	.757 (.024)	.749 (.023)	.728 (.021)	.749 (.021)
	vanilla	MM	.605 (.028)	.609 (.021)	.576 (.017)	.593 (.019)
		MICE-linear	.687 (.022)	.705 (.024)	.651 (.023)	.684 (.024)
		MissForest	.8 (.024)	.788 (.027)	.727 (.034)	.77 (.03)

Downstream classification task balanced accuracy (higher is better) for logistic regression (linear), support vector machine (SVM), single-layer perceptron (single layer), and multi-layer perceptron (MLP) models. Average and standard deviation across 20 AE training runs per configuration of mask encoding, loss normalization, and imputation mode. Best results per model are depicted in bold.

Table S5: Downstream length-of-stay regression task results

Mask encoded	Loss normalization	Imputation mode	linear	SVM	single layer	MLP
yes	observed-macro	MM	**2.199 (.074)**	1.874 (.084)	1.984 (.08)	**1.946 (.078)**
		MICE-linear	2.206 (.077)	1.878 (.08)	1.99 (.08)	1.951 (.072)
		MissForest	2.202 (.072)	1.875 (.082)	1.984 (.077)	1.947 (.076)
	observed-micro	MM	2.209 (.077)	1.876 (.085)	1.989 (.082)	1.95 (.079)
		MICE-linear	2.2 (.077)	1.876 (.085)	**1.982 (.082)**	1.949 (.078)
		MissForest	2.202 (.08)	**1.872 (.087)**	1.986 (.085)	1.95 (.082)
	vanilla	MM	2.207 (.077)	1.904 (.086)	1.994 (.086)	1.99 (.08)
		MICE-linear	2.237 (.077)	1.893 (.086)	2.019 (.085)	1.979 (.08)
		MissForest	2.297 (.078)	1.929 (.094)	2.067 (.087)	2.016 (.09)
no	observed-macro	MM	2.753 (.074)	2.072 (.087)	2.556 (.08)	2.372 (.092)
		MICE-linear	2.795 (.088)	2.121 (.09)	2.554 (.085)	2.386 (.091)
		MissForest	2.618 (.09)	2.136 (.099)	2.377 (.098)	2.284 (.098)
	observed-micro	MM	2.78 (.084)	2.077 (.09)	2.602 (.086)	2.424 (.088)
		MICE-linear	2.801 (.086)	2.125 (.088)	2.566 (.084)	2.46 (.096)
		MissForest	2.587 (.079)	2.117 (.094)	2.36 (.09)	2.268 (.092)
	vanilla	MM	2.789 (.082)	2.093 (.088)	2.634 (.087)	2.446 (.089)
		MICE-linear	2.753 (.088)	2.132 (.093)	2.531 (.093)	2.44 (.1)
		MissForest	2.4 (.103)	2.014 (.098)	2.167 (.111)	2.137 (.096)

Downstream regression task mean absolute error (lower is better) for linear regression (linear), support vector machine (SVM), single-layer perceptron (single layer), and multi-layer perceptron (MLP) models. Average and standard deviation across 20 AE training runs per configuration of mask encoding, loss normalization, and imputation mode. Best results per model are depicted in bold.

Table S6: Synthetic variable missing rate deviation

Loss normalization	Variable	MM	MICE-linear	MissForest
observed-macro	MAP	.037 (.008)	.036 (.006)	.033 (.005)
	DBP	.036 (.008)	.035 (.006)	.032 (.005)
	SBP	.035 (.008)	.034 (.006)	.032 (.005)
	urine output	.054 (.017)	.044 (.018)	.052 (.014)
	ALT	.02 (.003)	.02 (.003)	.019 (.002)
	AST	.02 (.003)	.02 (.002)	.019 (.002)
	pO2	.062 (.005)	.063 (.006)	.058 (.005)
	lactate	.089 (.006)	.088 (.006)	.084 (.007)
	creatinine	.14 (.007)	.136 (.007)	.135 (.007)
	GCS	.173 (.032)	.149 (.022)	.152 (.026)
	FiO2	.063 (.008)	.058 (.01)	.061 (.01)
	bolus	.025 (.004)	.026 (.003)	.025 (.002)
	vasopressor	.047 (.006)	.042 (.009)	.042 (.008)
observed-micro	MAP	.037 (.013)	.037 (.007)	.045 (.008)
	DBP	.036 (.012)	.036 (.008)	.044 (.008)
	SBP	.035 (.013)	.035 (.007)	.044 (.008)
	urine output	.066 (.013)	.065 (.015)	.064 (.01)
	ALT	.019 (.008)	.017 (.003)	.02 (.003)
	AST	.019 (.008)	.017 (.003)	.02 (.003)
	pO2	.06 (.012)	.058 (.007)	.063 (.009)
	lactate	.081 (.015)	.081 (.006)	.088 (.01)
	creatinine	.134 (.016)	.131 (.006)	.138 (.007)
	GCS	.168 (.036)	.155 (.018)	.178 (.027)
	FiO2	.063 (.008)	.056 (.01)	.063 (.009)
	bolus	.034 (.008)	.036 (.004)	.039 (.004)
	vasopressor	.049 (.014)	.049 (.008)	.043 (.012)
vanilla	MAP	.038 (.006)	.039 (.005)	.039 (.006)
	DBP	.037 (.006)	.039 (.005)	.038 (.006)
	SBP	.036 (.006)	.037 (.005)	.037 (.006)
	urine output	.073 (.01)	.066 (.014)	.063 (.011)
	ALT	.017 (.002)	.015 (.004)	.014 (.002)
	AST	.017 (.002)	.015 (.004)	.014 (.002)
	pO2	.054 (.005)	.046 (.005)	.044 (.004)
	lactate	.078 (.006)	.071 (.006)	.068 (.006)
	creatinine	.125 (.004)	.131 (.006)	.122 (.004)
	GCS	.102 (.007)	.099 (.007)	.122 (.01)
	FiO2	.051 (.006)	.048 (.006)	.052 (.008)
	bolus	.027 (.002)	.029 (.002)	.034 (.004)
	vasopressor	.03 (.008)	.034 (.008)	.023 (.011)

Absolute difference (lower is better) between synthetic an test set missing rate per variable. Average and standard deviation across 20 training runs per loss normalization and imputation method. Mask encoding is used for all configurations.

Table S7: Synthetic data distribution distances with encoded mask

Loss normalization	Metric	Variable	MM	MICE-linear	MissForest
observed-macro	Wasserstein distance	MAP	.295 (.009)	.296 (.009)	.295 (.014)
		DBP	.295 (.008)	.294 (.011)	.293 (.013)
		SBP	.288 (.008)	.287 (.013)	.286 (.013)
		urine output	.23 (.02)	.242 (.029)	.247 (.017)
		ALT	.127 (.027)	.114 (.031)	.15 (.022)
		AST	.148 (.029)	.131 (.035)	.157 (.022)
		pO2	.285 (.022)	.31 (.021)	.326 (.024)
		lactate	.342 (.029)	.344 (.038)	.349 (.031)
		creatinine	.136 (.023)	.142 (.021)	.139 (.02)
	Mean absolute class ratio difference	GCS	.009 (.002)	.008 (.001)	.008 (.001)
		FiO2	.008 (.002)	.009 (.002)	.009 (.002)
		bolus	.026 (.014)	.033 (.014)	.033 (.009)
		vasopressor	.019 (.008)	.024 (.01)	.024 (.006)
observed-micro	Wasserstein distance	MAP	.23 (.015)	.232 (.008)	.222 (.014)
		DBP	.237 (.019)	.239 (.01)	.23 (.013)
		SBP	.232 (.015)	.233 (.01)	.222 (.014)
		urine output	.248 (.011)	.26 (.014)	.272 (.015)
		ALT	.365 (.032)	.37 (.016)	.479 (.018)
		AST	.353 (.033)	.361 (.018)	.462 (.02)
		pO2	.458 (.018)	.457 (.019)	.47 (.017)
		lactate	.426 (.033)	.422 (.016)	.473 (.019)
		creatinine	.406 (.016)	.427 (.016)	.5 (.015)
	Mean absolute class ratio difference	GCS	.01 (.001)	.009 (.001)	.009 (.001)
		FiO2	.009 (.005)	.01 (.002)	.01 (.002)
		bolus	.07 (.014)	.101 (.018)	.188 (.015)
		vasopressor	.022 (.008)	.019 (.007)	.017 (.007)
vanilla	Wasserstein distance	MAP	.381 (.013)	.383 (.01)	.376 (.019)
		DBP	.375 (.011)	.378 (.012)	.374 (.017)
		SBP	.373 (.011)	.37 (.009)	.369 (.015)
		urine output	.66 (.017)	.498 (.012)	.45 (.018)
		ALT	.763 (.013)	.744 (.017)	.622 (.043)
		AST	.771 (.013)	.712 (.014)	.557 (.043)
		pO2	.708 (.007)	.6 (.009)	.536 (.02)
		lactate	.741 (.008)	.517 (.022)	.521 (.028)
		creatinine	.757 (.01)	.656 (.01)	.566 (.039)
	Mean absolute class ratio difference	GCS	.037 (.002)	.025 (.001)	.02 (.004)
		FiO2	.039 (.003)	.019 (.003)	.094 (.009)
		bolus	.142 (.014)	.111 (.012)	.115 (.03)
		vasopressor	.042 (.006)	.054 (.007)	.035 (.012)

Distance metrics between synthetic data and test set per variable. Average and standard deviation across 20 training runs per loss normalization and imputation method. Mask encoding is used for all configurations. Distance is measured as Wasserstein distance (lower is better) for metric variables and mean absolute difference in class ratios (lower is better) for ordinal class variables.

Table S8: Synthetic data distribution distances without encoded mask

Loss normalization	Metric	Variable	MM	MICE-linear	MissForest
observed-macro	Wasserstein distance	MAP	.322 (.007)	.318 (.008)	.315 (.007)
		DBP	.32 (.004)	.32 (.009)	.309 (.007)
		SBP	.316 (.01)	.312 (.01)	.307 (.01)
		urine output	.314 (.012)	.305 (.012)	.305 (.01)
		ALT	.389 (.026)	.369 (.024)	.486 (.021)
		AST	.399 (.023)	.38 (.024)	.468 (.022)
		pO2	.474 (.01)	.505 (.013)	.614 (.012)
		lactate	.486 (.018)	.516 (.019)	.665 (.02)
		creatinine	.177 (.016)	.206 (.021)	.399 (.017)
	Mean absolute class ratio difference	GCS	.013 (.002)	.01 (.002)	.008 (.001)
		FiO2	.035 (.002)	.041 (.002)	.04 (.002)
		bolus	.322 (.004)	.282 (.011)	.196 (.012)
		vasopressor	.1 (.008)	.113 (.007)	.132 (.009)
observed-micro	Wasserstein distance	MAP	.258 (.009)	.256 (.007)	.254 (.009)
		DBP	.264 (.01)	.26 (.008)	.253 (.01)
		SBP	.254 (.01)	.253 (.005)	.242 (.008)
		urine output	.335 (.011)	.326 (.012)	.326 (.013)
		ALT	.496 (.019)	.51 (.032)	.65 (.017)
		AST	.484 (.016)	.481 (.03)	.622 (.014)
		pO2	.59 (.014)	.58 (.016)	.63 (.018)
		lactate	.553 (.019)	.511 (.023)	.595 (.027)
		creatinine	.438 (.015)	.473 (.023)	.55 (.013)
	Mean absolute class ratio difference	GCS	.014 (.001)	.011 (.002)	.009 (.002)
		FiO2	.031 (.002)	.038 (.002)	.039 (.003)
		bolus	.207 (.008)	.166 (.009)	.294 (.01)
		vasopressor	.177 (.004)	.11 (.007)	.109 (.013)
vanilla	Wasserstein distance	MAP	.422 (.012)	.416 (.013)	.373 (.051)
		DBP	.424 (.01)	.417 (.012)	.373 (.045)
		SBP	.418 (.015)	.408 (.012)	.375 (.047)
		urine output	.71 (.008)	.572 (.012)	.461 (.046)
		ALT	.806 (.012)	.798 (.013)	.679 (.083)
		AST	.816 (.012)	.778 (.012)	.602 (.075)
		pO2	.773 (.006)	.714 (.006)	.638 (.04)
		lactate	.787 (.007)	.666 (.01)	.654 (.059)
		creatinine	.79 (.007)	.693 (.015)	.533 (.076)
	Mean absolute class ratio difference	GCS	.056 (.004)	.054 (.006)	.018 (.006)
		FiO2	.119 (.005)	.047 (.005)	.095 (.01)
		bolus	.361 (.004)	.303 (.013)	.129 (.047)
		vasopressor	.363 (.007)	.22 (.01)	.093 (.012)

Distance metrics between synthetic data and test set per variable. Average and standard deviation across 20 training runs per loss normalization and imputation method. Mask encoding is never used. Distance is measured as Wasserstein distance (lower is better) for metric variables and mean absolute difference in class ratios (lower is better) for ordinal class variables.
